# Characteristics of *Salvia miltiorrhiza* methylome and the regulatory mechanism of DNA methylation in tanshinone biosynthesis

**DOI:** 10.1093/hr/uhad114

**Published:** 2023-05-31

**Authors:** Jiang Li, Caili Li, Yuxing Deng, Hairong Wei, Shanfa Lu

**Affiliations:** Key Lab of Chinese Medicine Resources Conservation, State Administration of Traditional Chinese Medicine of the People' s Republic of China, Institute of Medicinal Plant Development, Chinese Academy of Medical Sciences & Peking Union Medical College, Beijing 100193, China; Engineering Research Center of Chinese Medicine Resource, Ministry of Education, Beijing 100193, China; Key Lab of Chinese Medicine Resources Conservation, State Administration of Traditional Chinese Medicine of the People' s Republic of China, Institute of Medicinal Plant Development, Chinese Academy of Medical Sciences & Peking Union Medical College, Beijing 100193, China; Engineering Research Center of Chinese Medicine Resource, Ministry of Education, Beijing 100193, China; Key Lab of Chinese Medicine Resources Conservation, State Administration of Traditional Chinese Medicine of the People' s Republic of China, Institute of Medicinal Plant Development, Chinese Academy of Medical Sciences & Peking Union Medical College, Beijing 100193, China; Engineering Research Center of Chinese Medicine Resource, Ministry of Education, Beijing 100193, China; College of Forest Resources and Environmental Science, Michigan Technological University, Houghton, MI 49931, USA; Key Lab of Chinese Medicine Resources Conservation, State Administration of Traditional Chinese Medicine of the People' s Republic of China, Institute of Medicinal Plant Development, Chinese Academy of Medical Sciences & Peking Union Medical College, Beijing 100193, China; Engineering Research Center of Chinese Medicine Resource, Ministry of Education, Beijing 100193, China

## Abstract

*Salvia miltiorrhiza* is a model medicinal plant with significant economic and medicinal value. Its roots produce a group of diterpenoid lipophilic bioactive components, termed tanshinones. Biosynthesis and regulation of tanshinones has attracted widespread interest. However, the methylome of *S. miltiorrhiza* has not been analysed and the regulatory mechanism of DNA methylation in tanshinone production is largely unknown. Here we report single-base resolution DNA methylomes from roots and leaves. Comparative analysis revealed differential methylation patterns for CG, CHG, and CHH contexts and the association between DNA methylation and the expression of genes and small RNAs. Lowly methylated genes always had higher expression levels and 24-nucleotide sRNAs could be key players in the RdDM pathway in *S. miltiorrhiza*. DNA methylation variation analysis showed that CHH methylation contributed mostly to the difference. Go enrichment analysis showed that diterpenoid biosynthetic process was significantly enriched for genes with downstream overlapping with hypoCHHDMR in July_root when comparing with those in March_root. Tanshinone biosynthesis-related enzyme genes, such as *DXS2*, *CMK*, *IDI1*, *HMGR2*, *DXR*, *MDS*, *CYP76AH1, 2OGD25*, and *CYP71D373,* were less CHH methylated in gene promoters or downstream regions in roots collected in July than those collected in March. Consistently, gene expression was up-regulated in *S. miltiorrhiza* roots collected in July compared with March and the treatment of DNA methylation inhibitor 5-azacytidine significantly promoted tanshinone production. It suggests that DNA methylation plays a significant regulatory role in tanshinone biosynthesis in *S. miltiorrhiza* through changing the levels of CHH methylation in promoters or downstreams of key enzyme genes.

## Introduction

Cytosine DNA methylation, which forms 5mC through introducing a methyl group to the fifth carbon of a cytosine residue, is a dominating epigenetic modification mechanism widely existed and highly conserved in eukaryotes [1, 2]. It has species, tissues and organs specificity and plays vital regulatory roles in many aspects of eukaryotes, such as transposon silencing, genomic imprinting, and X chromosome inactivation [2]. In plants, through affecting transposon mobility, regulating gene expression, and modulating chromatin structure, DNA methylation contributes to evolution, photosynthesis, development, and response to abiotic (e.g., salinity, drought, and heat) and biotic stresses (e.g., viruses, bacteria, and fungi) [3–7]. Generally, methylation at gene promoter regions will improve the binding of transcription repressors and prevent the binding of transcription activators, leading to reduced or inactivated transcription, whereas methylation at coding regions will inhibit transcript elongation, resulting in smaller and possibly inactive transcripts [3, 6, 8]. It can also activate gene transcription through currently unknown mechanisms [3].

DNA methylation in plants occurs at specific cytosine DNA sequence contexts. It includes CG and non-CG. The latter can be further divided into CHG and CHH, where H can be A, T, or C. DNA methylation is dynamic, involving *de novo* methylation (also known as establishment), maintenance, and demethylation. Establishment is catalyzed by domain-rearranged methltransferases (DRMs) through RNAi-dependent and -independent pathways. There is crosstalk between non-CG methylation (non-mCG) and CG methylation (mCG) [4, 9]. In the RNAi-dependent pathway, RNA-directed DNA methylation (RdDM) is involved. During RdDM, DRM2 is guided to the target locus and then directs methylation via 21–22-nucleotide (non-canonical RdDM) or 24-nucleotide (canonical RdDM) small RNAs (sRNAs) [2, 9, 10]. In the RNAi-independent pathway, chromo-methyltransferases (CMTs), instead of DRM2, act as *de novo* methyltransferases [11, 12]. Differently from *de novo* DNA methylation showing crosstalk between mCG and non-mCG, methylation maintenance of three sequence contexts is almost independent. mCG, CHG methylation (mCHG) and CHH methylation (mCHH) are maintained by DNA methyltransferase 1 (MET1), chromo-methyltransferase 2 or 3 (CMT2/CMT3), and DRM2 or CMT2, respectively [4, 13]. In addition, the introduced methyl group can be removed through active or passive demethylation. The former is catalyzed by DEMETER-like DNA glyscosylases in combination with the base excision repair pathway [14]. It leads to replacement of the methylated cytosine with nonmethylated. The latter is caused by dysfunction of MET1 and occurs in newly synthesized DNA strand [2].

With the advances in sequencing technologies, single base pair resolution methylomes have been reported in *Arabidopsis* and many other plant species [15–20]. Analysis of plant DNA methylomes showed that some of methylation features were highly conserved among species. For instance, mCG was generally the most abundant and mCG level was the highest [18, 21], and all sequence contexts were highly methylated in the transposon and repeat regions [15, 20, 22, 23]. On the other hand, variations were widely found among different plant methylomes [24]. For example, in comparison with other plant species examined, *Arabidopsis* had lower mCG, whereas beet (*Beta vulgaris*) had higher mCHH [24]. In maize, mCHH islands existing in gene flanking regions were positively correlated with gene expression. However, the phenomenon was not universal in other plant species [17, 24]. DNA methylome variation seems to be important in regulating seed development [25], stem and leaf growth [26, 27], vernalization [28], fruit ripening [29, 30], and secondary metabolism [31–33].

*Salvia miltiorrhiza* is a well-known medicinal plant species. It belongs to the genus *Salvia* of the Lamiaceae family and has been used for more than two thousand years in treating diseases, such as amenorrhoea, dysmenorrhea, heart diseases, and cardiovascular diseases [34]. In recent years, with a huge number of transcriptome and sRNAome data available and the sequencing of whole genome, *S. miltiorrhiza* has attracted widespread interest and become a model medicinal plant [34–38].

Tanshinones are a group of diterpenoid lipophilic components. It mainly distributed in root periderm of *S. miltiorrhiza* [39]. Tanshinone biosynthesis is mainly through the 2-C-methyl-D-erythritol 4-phosphate (MEP) pathway located in the plastid in crosstalk with the mevalonate (MVA) pathway in the cytoplasm, followed by the formation of geranylgeranyl diphosphate (GGPP) and parent carbon skeleton miltiradiene [34]. At least 26 enzyme genes, such as *SmIDI1*, *SmCPS1*, *SmKSL1*, *SmGGPPS1*, *SmCYP76AH1*, *SmCYP76AH3*, *SmCYP76AK1*, *SmCYP71D373*, *SmCYP71D375*, *SmCYP411*, and *SmTIIAs*, are involved in tanshinone biosynthesis [40–43]. Previous studies showed that tanshinone biosynthesis could be regulated by DNA methylation [44–46]. However, the underlying mechanism is unknown. Here, we generated and comparatively analysed single-base resolution maps of DNA methylation in *S. miltiorrhiza* roots collected in July and March and leaves collected in July. Tanshinone production in *S. miltiorrhiza* hairy roots treated with DNA methylation inhibitor 5-azacytidine was also analyzed. Our results reveal the correlation between tanshinone accumulation and methylation level of key enzyme genes and suggest the significance of CHH methylation level in regulating tanshinone biosynthesis.

## Results

### Genes involved in DNA methylation pathway and global DNA methylation patterns in *S. miltiorrhiza*

To reveal the features and regulatory roles of DNA methylation in *S. miltiorrhiza*, we first analysed genes involved in DNA methylation pathway in *S. miltiorrhiza* through BLAST search of *Arabidopsis* DNA methylation pathway-related genes against the whole genome of *S. miltiorrhiza* line 99–3 [36]. It resulted in the identification of 39 genes, of which 12 were previously reported [44, 45], whereas the other 26 were novel (Table 1). Further transcriptome analysis showed that, except *MORC6*, all DNA methylation pathway-related genes were expressed in *S. miltiorrhiza* (Table 1). It suggests the conservation of DNA methylation pathway in *S. miltiorrhiza*.

**Table 1:** Putative DNA methylation pathway genes in *S. miltiorrhiza*.

	Name (*Arabidopsis*)	Length (aa)	*Salvia miltiorrhiza* orthologs	Expression level (FPKM)
MET1	VIM1,2,3,4,5,6	763	SMil_00019061	18.517382
	MET1,2a,2b,3	1677	MG602207^*^	3.812192
CMT3	SUVH4	719	SMil_00020483	2.142947
	CMT2	957	MG602209^*^, MG602210^*^	3.035007, 1.942858
	CMT3	819	MG602208^*^	1.276837
Pol IV recruit	CLSY1/CLSY2	485	SMil_00014592	10.323617
	SHH1/SHH2	295	SMil_00020772	49.920265
Pol IV	NRPD1	1360	SMil_00023978	5.904174
Pol IV + V	NRPD2/NRPE2	1217	SMil_00015068	33.532993
Pol IV + V	NRPD4/NRPE4	221	SMil_00017112	14.302505
Pol V	NRPE1	2414	SMil_00019050	5.564985
Pol V	NRPE5	228	SMil_00007967	13.797972
Pol V	NRPE9B	247	SMil_00024808	2.925006
Pol V recruit	DRD1	922	SMil_00009680	13.565602
	DMS3	433	SMil_00007199	11.484639
	RDM1	168	SMil_00010839	6.756174
	SUVH2/9	627	SMil_00026658	7.278948
RdDM	RDR2	972	KF872206^*^	7.845572
	DCL1	1927	KF366499^*^	20.077427
	DCL2	1385	KF366500^*^	15.568172
	DCL3	1654	KF366501^*^	9.305597
	DCL4	1628	KF366502^*^, KF366503^*^	8.573687, 18.415268
	HEN1	872	SMil_00012171	6.005173
	AGO4	870	KF153682^*^	89.73568
	KTF1	1488	SMil_00015344	4.421493
	IDN2	774	SMil_00009894	8.423001
	SUVR2	441	SMil_00015021	10.132948
	DMS4	364	SMil_00008543	19.366898
	UBP26	1097	SMil_00015412	28.874403
	DRM2	689	MG602213^*^	4.019984
	LDL1	818	SMil_00009947	6.830226
	LDL2	1149	SMil_00008094	5.881756
	JMJ14	839	SMil_00011756	8.693495
Others	HDA6	376	SMil_00017396	28.999033
	RDR6	1195	KF872205^*^	59.507931
	MORC6	701	SMil_00022541	0
	DDM1	705	SMil_00012788	2.728436

Length (aa) shows the longest protein analysed. * This *S. miltiorrhiza* ortholog could be found in NCBI database and is from our previous study.

We then generated single-base resolution maps of DNA methylation for field-grown *S. miltiorrhiza* roots collected in March before tanshinone accumulation and in July when tanshinone rapidly accumulated [47]. In addition, leaves collected from *S. miltiorrhiza* plants in July were also analysed. Hereafter, they were referred to as March_root, July_root, and July_leaf, respectively. A total of 84 836 367, 66 748 243, and 65 818 905 paired**-**end raw reads with 150 bp in length were obtained for March_root, July_root, and July_leaf, respectively (Table S1, see online supplementary material). Lambda genome was used to calculate bisulfite conversion rate. The results showed that they were above 99.5% for all three samples, suggesting a high bisulfite conversion rate (Table S1, see online supplementary material). After strict quality control processing and reads mapping, approximately 50% of clean reads were uniquely mapped, covering >87% of the genome. All three sequenced methylomes yielded an average coverage depth of >14.94× in the genome and about 80% of cytosines had at least one read (Fig. S1, see online supplementary material). These results indicated that BS-Seq data was sufficient for DNA methylation analysis at single cytosine resolution.

**Figure 1:**
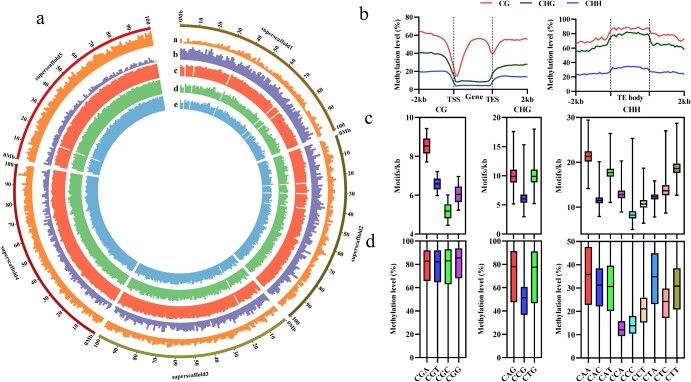
DNA methylation landscape of *S. miltiorrhiza*. **a** DNA methylation landscape depicted using 1-Mb-wide bins across superscaffolds in March_root. Units on the circumference show megabase values. Track a, TE coverage (5%–18% per Mb). Track b, gene density (4–89 per Mb). Track c, the level of CG methylation (1.6%–87% per Mb). Track d, the level of CHG methylation (1.2%–77.5% per Mb). Track e, the level of CHH methylation (0.7%–32.6% per Mb). **b** The levels of DNA methylation of CG, CHG, and CHH for genes and TEs. **c** Densities of each sub-contexts across scaffolds. **d** The levels of DNA methylation of each sub-contexts across scaffolds.

To facilitate methylation analysis, *S. miltiorrhiza* line 99–3 genome was constructed into six superscaffolds through connecting 21 045 smaller scaffolds. Global landscapes of DNA methylation of superscaffold1 to superscaffold5 were displayed in Fig. 1a, as shown in which transposable element (TE) coverage was positively correlated with the level of DNA methylation in three sequence contexts. In contrast, the density of genes showed negative correlation (Fig. 1a). Detailed analysis of DNA methylation levels in TE and genic regions showed that the methylation levels of TEs were obviously higher than that of genic regions (Fig. 1b), confirming the observation in Fig. 1a. mCG in genic regions showed a reduction in transcriptional start/end sites but an increase in gene body and flanking regions. mCHH and mCHG were elevated in flanking regions but relatively depleted in gene bodies (Fig. 1b). Methylation levels of all sequence contexts were higher in TE body than those in the upstream or downstream regions (Fig. 1b). Analysis of single site methylation showed that mCG and mCHG had bimodal distribution patterns for each cytosine (Fig. S2, see online supplementary material), implying a robust maintenance system for mCG and mCHG. Differently, most CHH sites were either not methylated or methylated at relatively low levels (Fig. S2, see online supplementary material).

Further analysis of mC in the *S. miltiorrhiza* genome showed that the four CG sub-contexts had comparable methylation levels (Fig. 1d), although they exhibited different densities (Fig. 1c). Among the CHG sub-contexts, CCG had lower density and methylation level than CAG and CTG (Fig. 1c and d). Among the CHH sub-contexts, the density and methylation level of CAA were the highest, and CCH (CCC, CCA, and CCT) had lower methylation levels in comparison with CAH (H = A, T, or C) and CTH (Fig. 1c and d).

### Effects of DNA methylation on gene expression

**Figure 2:**
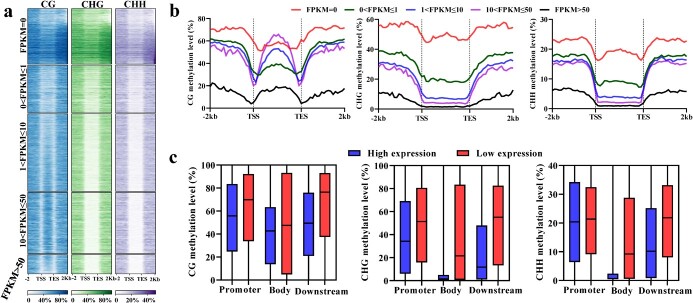
The effects of DNA methylation on gene expression. **a** The levels of DNA methylation across gene bodies and the 2 kb flanking regions. Based on expression levels, genes were divided into five groups. For methylation calculation, gene bodies and the flanking regions were equally divided into 20 bins, respectively. **b** The average DNA methylation of five gene groups in CG, CHG, and CHH sequence contexts. **c** Comparison of DNA methylation between the lowly and the highly expressed genes for each genic region. One-third of the genes with the highest expression levels were defined as highly expressed, whereas one-third of the genes with the lowest expression levels was defined as lowly expressed. Promoter, upstream 2 kb. Downstream, downstream 2 kb.

To learn the relationship between gene expression and DNA methylation, we divided genes into five groups based on the levels of expression. The results showed a reverse correlation between gene expression and DNA methylation, but not between gene body mCG and transcription (Fig. 2a). It suggests the regulatory roles of DNA methylation in gene expression and implies the complexity between DNA methylation and gene expression. The heaviest gene body mCG was observed in moderately highly expressed genes (10 < FPKM ≤50) (Fig. 2a). It is consistent with previous studies on other plants [16, 48]. Moreover, in the promoters and the downstream regions, genes with higher expression showed lower mCG levels, whereas in gene body regions, the most highly expressed (FPKM >50) and relatively low expressed (0 < FPKM ≤1) genes had the lowest and relatively low mCG, respectively (Fig. 2b). It implies the difference of mCG-involved transcription regulation in flanking regions and gene body regions. For mCHG and mCHH, lower methylation levels were found for all three genic regions (gene promoter, downstream, and body) of higher expressed genes (Fig. 2b). In order to further analyse the roles of DNA methylation, genes were grouped into highly and lowly expressed groups. Methylation levels were compared in different genomic regions. The results showed that, regardless of genomic regions, lowly expressed genes had a higher degree of DNA methylation levels for all sequence contexts (Fig. 2c). These data suggest that, in *S. miltiorrhiza*, DNA methylation generates functional constraints on gene expression.

### Correlation between sRNA distribution and DNA methylation

**Figure 3:**
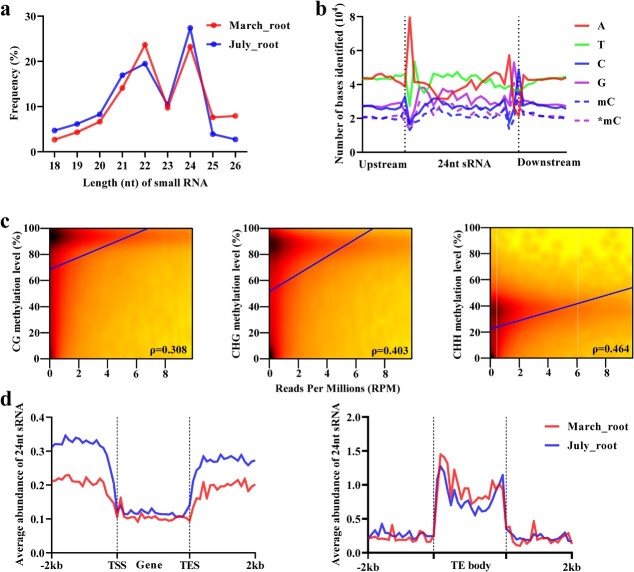
Association between DNA methylation and 24-nucleotide sRNA. **a** Length size distribution of small RNAs in March_root and July_root. **b** Nucleotide frequency distribution of 24-nucleotide sRNA mapping and 10-nucleotide flanking regions. mC was defined as methylated cytosine on the sense strand; mC* was defined as methylated cytosines on the antisense strand. **c** The correlation between DNA methylation and 24-nucleotide sRNA abundance. ρ: Spearman’s rank correlation coefficient (*P*-value <0.001). **d** The average abundance (RPM) distribution of 24-nucleotide sRNAs across gene/TE body and flanking 2 kb regions in March_root and July_root.

In *Arabidopsis*, some 21–22-nucleotide and 24-nucleotide sRNAs are involved in RdDM pathways that lead to *de novo* DNA methylation at genomic regions with sequences complementary to those sRNAs [2, 9, 10]. To investigate the role of *S. miltiorrhiza* sRNAs in DNA methylation, we sequenced the sRNAome in March_root and July_root using high-throughput sequencing technology. After quality control and adapter trimming, a total of 12 126 660 and 11 303 818 clean reads were obtained in March_root and July_root, respectively. 21-, 22- and 24-nucleotide sRNAs were the three most abundant in two tissues (Fig. 3a). It is consistent with the previous observation of sRNAs from replanting *S. miltiorrhiza* plants [49].

Next, we analysed the relevance between the cytosine and guanine of sRNAs and the cytosine methylation state of their complementary DNA sequences in *S. miltiorrhiza*. If *S. miltiorrhiza* sRNAs direct DNA methylation through the RdDM pathway, correlation will be found between them [20]. The 21- to 24-nucleotide unique sequences were mapped to the genome of *S. miltiorrhiza* line 99–3. Then, sRNA base composition, the number of methylated cytosines in the sRNA-mapped sites and the 10-bp flanking regions (denoted by mC) and those in the corresponding complementary DNA strand (denoted by *mC) were analysed. The results showed that the number of mC and *mC displayed close correlation with cytosine and guanine number from 24-nucletide sRNAs, respectively (Fig. 3b). However, this pattern was not observed for 21- to 23-nucleotide sRNAs (Fig. S3, see online supplementary material). The results were consistent with those from castor bean seeds [20], implying the conservation and significance of 24-nucleotide sRNAs in DNA methylation in plants.

Finally, we analysed correlation between 24-nucletide sRNAs and context specific methylation levels within 2000-bp bins throughout the genome. The highest correlation was observed between CHH DNA methylation and 24-nt sRNA (Fig. 3c). Distribution pattern analysis showed that *S. miltiorrhiza* 24-nucleotide sRNAs were preferentially located in the promoters and the 3′ flanking regions of genes (Fig. 3d). Their distribution in the 5′ and 3′ flanking regions of TE was comparable with the promoters and the 3′ flanking regions of genes, whereas in the TE body regions, the distribution of 24-nucleotide sRNAs was much higher (Fig. 3d). Interestingly, 24-nt sRNA of genic regions displayed higher abundance in July_root than March_root, whereas TE regions showed no obvious difference (Fig. 3d). Taken together, 24-nucleotide sRNAs could be key players in the RdDM pathway in *S. miltiorrhiza*.

### Characterization of DNA methylation variation among different *S. miltiorrhiza* samples

**Figure 4:**
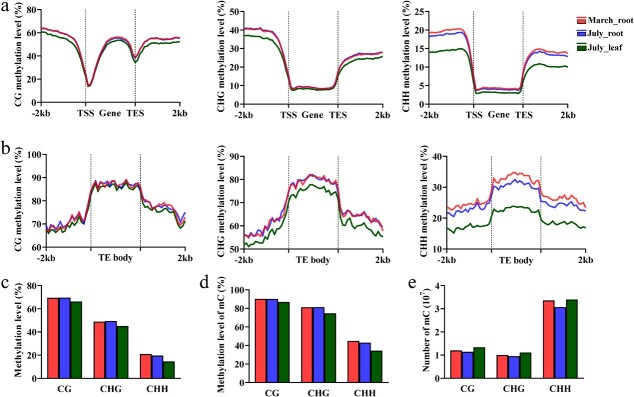
Genome-wide comparative analysis of DNA methylation in *S. miltiorrhiza*. **a** DNA methylation levels in CG, CHG, and CHH sequence contexts for gene body and flanking regions in three samples. **b** DNA methylation levels in CG, CHG, and CHH sequence contexts for TE body and flanking regions in three samples. **c** Global methylation levels in all sequence contexts in three samples. **d** Global methylation levels of mC in all sequence contexts in three samples. **e** Histograms showing the numbers of mCs of all three sequence contexts in three samples.

We calculated the average DNA methylation levels of gene and TE regions in three samples, including March_root, July_root, and July_leaf. In the two root samples, symmetrical sequence contexts (CG or CHG) had comparable methylation levels in both gene and TE regions, whereas mCHH levels were lower in July_root than those in March_root (Fig. 4a and b). It implies the importance of mCHH in *S. miltiorrhiza* roots. Compared with those in July_root, methylation levels in gene and TE regions were lower in July_leaf, which is particularly true for the mCHH sequence context (Fig. 4a and b). Similar trends were observed when global methylation level was calculated (Fig. 4c). Considering the negative relationship between gene expression and DNA methylation (Fig. 2), these results imply that gene expression in July_leaf could be generally more active than that in *S. miltiorrhiza* roots.

In order to characterize the difference of DNA methylation among samples, we identified differentially methylated cytosines (DMCs) and differentially methylated regions (DMRs) (Table S2, see online supplementary material). The results showed that CG or CHG had almost the same amount of hyper and hypoDMC/DMRs in March_root and July_root; however, CHH hypoDMC/DMRs were significantly more than hyperDMC/DMRs in July_root (Fig. S4, see online supplementary material). In addition, all three sequence contexts showed more hyper DMC/DMRs in July_root than July_leaf, and an overwhelming number was from hyperCHH context (Fig. S5, see online supplementary material). These results were consistent with those from the comparative analysis of DNA methylation levels in the three *S. miltiorrhiza* samples (Fig. 4). It further confirms that the difference of DNA methylation is mostly contributed by mCHH.

Because DNA methylation difference could be resulted from methylation level variance of existing mC or number variance of total mCs, we comparatively analysed DNA methylation levels and numbers of mC in the three *S. miltiorrhiza* samples. The results showed that the methylation level and the number of mCHHs were decreased in July_root in comparison with those in March_root, whereas they were similar for mCG and mCHG (Fig. 4d and e). It suggests that both the methylation level of mCHH and the number of mCHH contributed to the difference of DNA methylation between March_root and July_root. In addition, methylation levels of mCs in all three sequence contexts were higher in July_root than July_leaf (Fig. 4d), whereas the number of mCs was slightly lower in July_root (Fig. 4e). It suggests that the difference of DNA methylation between July_root and July_leaf was contributed by methylation level of mC, but not mC number.

### Involvement of DNA methylation in biosynthesis and metabolism of terpenes

*S. miltiorrhiza* is a well-known medicinal plant species enriched in secondary metabolites [34]. So far, more than 200 compounds have been identified from this species [50, 51]. They include terpenoids, phenolic acids, flavonoids, quinones, and other compounds [34]. Thus, it is significant to know whether DNA methylation is involved in the regulation of secondary metabolism in *S. miltiorrhiza*. To address this question, comparative analysis of DNA methylation in March_root and July_root were firstly performed for the identification of DMR-related genes that have DMR location overlapping with gene bodies, promoters (2 kb) or 3′ flanking regions. For gene promoters overlapping with DMRs, a total of 747 hyperDMR- and 793 hypoDMR-related genes, 840 hyperDMR- and 784 hypoDMR-related genes, and 2166 hyperDMR- and 4319 hypoDMR-related genes were found for mCG, mCHG, and mCHH in July_root compared with March_root, respectively (Table S3, see online supplementary material). For gene bodies overlapping with DMRs, 746 hyperCGDMR- and 906 hypoCGDMR-related genes, 1039 hyperCHGDMR- and 1014 hypoCHGDMR-related genes, and 1315 hyperCHHDMR- and 2558 hypoCHHDMR-related genes were obtained (Table S4, see online supplementary material). In 3′ flanking regions, the numbers of hyperDMR-/hypoDMR-related genes for mCG, mCHG, and mCHH in July_root were 746/829, 817/672, and 1774/3570, respectively (Table S5, see online supplementary material). It suggests that symmetric sequence context (CG/CHG) has a similar number of hyperDMR- and hypoDMR-related genes, and mCHH has the highest number of DMR-related genes and more hypoDMR-related genes. It indicates the significance of mCHH in regulating gene expression in *S. miltiorrhiza*. Consistent with an overwhelming number of hyperDMRs in July_root compared with July_leaf, DMR-related genes were almost from hyperDMRs. In promoter regions, 970, 1068, and 9144 hyperDMR-related genes were found for mCG, mCHG, and mCHH, respectively. However, only 322, 311, and 827 hypoDMR-related genes were obtained for the three sequence contexts, respectively (Table S3, see online supplementary material). In gene body regions, 1001 hyperDMR- and 310 hypoDMR-related genes, 1278 hyperDMR- and 308 hypoDMR-related genes, and 4921 hyperDMR- and 484 hypoDMR-related genes were originated from CG, CHG, and CHH contexts, respectively (Table S4, see online supplementary material). In gene downstreams, the number of hyperDMR-/hypoDMR-related genes from mCG, mCHG, and mCHH were 919/293, 1136/246, and 8023/599, respectively (Table S5, see online supplementary material).

**Figure 5:**
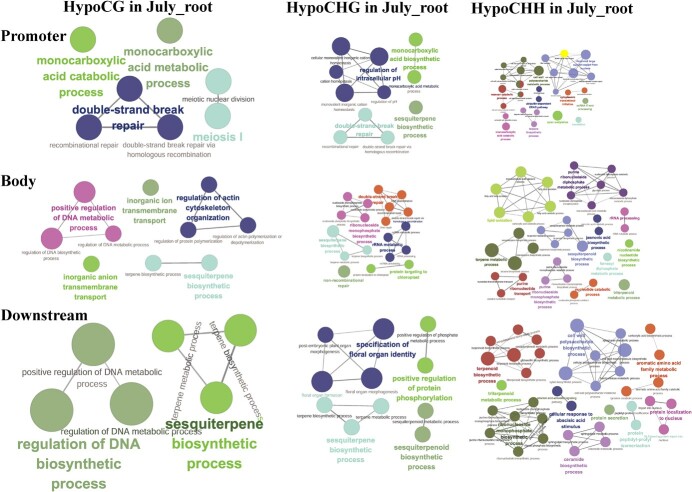
Biological processes significantly enriched in hypoDMR-related genes in July_root compared with March_root. Promoter presents genes with promoters overlapping with hypoDMR. Body represents genes with gene bodies overlapping with hypoDMR. Downstream represents genes with genes downstream overlapping with hypoDMR.

In order to preliminarily elucidate the potential functions of hyperDMR- and hypoDMR-related genes in promoter, gene body, and gene downstream, GO enrichment analysis was performed. The results showed that terpene biosynthesis and metabolism-related genes were significantly enriched for hypoDMR-related genes in July_root when comparing with those in March_root (Fig. 5), whereas more basic metabolism-related biological processes, such as cellular amino acid metabolic process and mitochondrial mRNA processing, were significantly enriched in hyperDMR-related genes (Fig. S6, see online supplementary material). The results are consistent with the facts that the biosynthesis and metabolism of terpenes are more active in fast-growing roots of *S. miltiorrhiza* plants than the roots collected in March [47], which was further confirmed by higher tanshinone contents in July_root than in March_root (Fig. S7, see online supplementary material). Because apparent DNA hypermethylation in July_root compared with July_leaf, we performed GO analysis for hyperDMR-related genes for three sequence contexts in different gene regions (Fig. S8, see online supplementary material). Genes involved in leaf development, chlorophyll biosynthetic and metabolic processes are highly enriched for CG context in gene downstream. For other context-specific DMR-related genes in different genomic positions, genes performing cellular amino acid biosynthetic process, DNA repair, mRNA processing, and carbohydrate biosynthetic process were significantly enriched. It suggests that DNA methylation could be an important regulatory factor for development and biological functions of leaves in *S. miltiorrhiza*.

**Figure 6:**
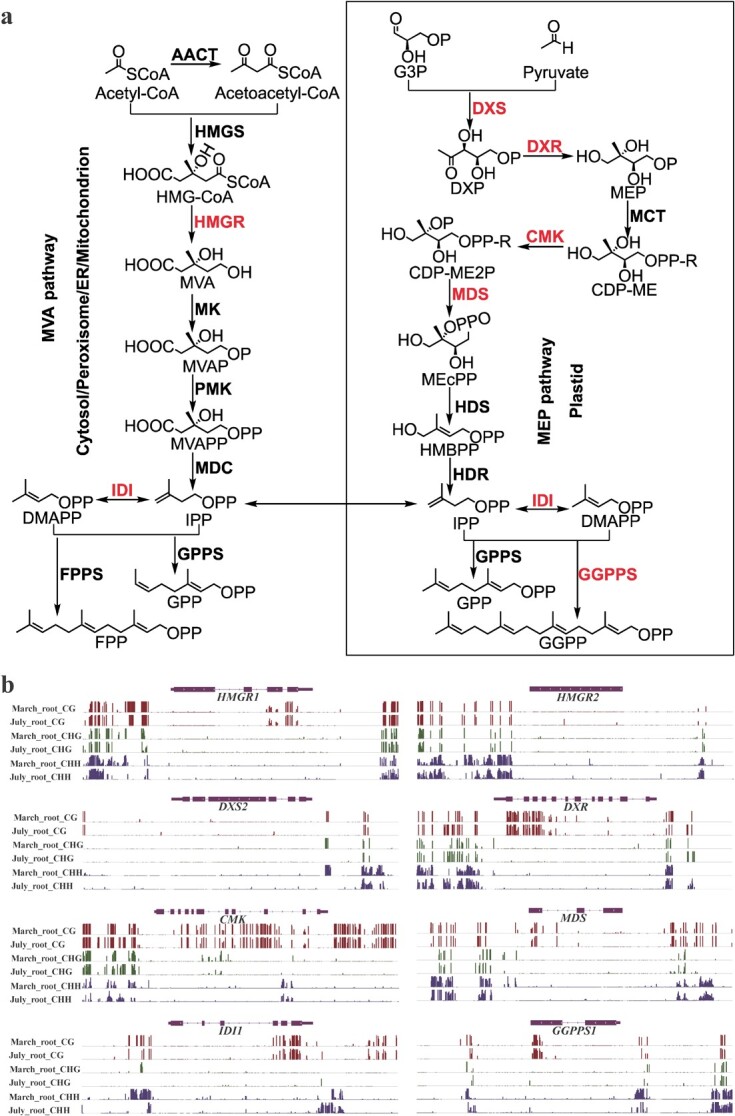
DMR-related enzyme genes in the upstream pathway of tanshinone biosynthesis. **a** The upstream pathway of tanshinone biosynthesis [34]. It includes the MEP pathway located in the plastid, the MVA pathway located in the cytosol, endoplasmic reticulum (ER), peroxisome and mitochondrion, and the biosynthesis of intermediate diphosphate precursors. Among them, the MEP pathway synthesizes intermediates 1-deoxy-D-xyulose 5-phosphate (DXP), 2-C-methyl-D-erythritol 4-phosphate (MEP), 4-diphosphocytidyl-2-C-methyl-D-erythritol (CDP-ME), 4-diphosphocytidyl-2-C-methyl-D-erythritol 2-phosphate (CDP-ME2P), 2-C-methyl-D-erythritol 2,4-cyclodiphosphate (MEcPP), 1-hydroxy-2-methyl-2-(E)-butenyl 4-diphosphate (HMBPP), and isopentenyl diphosphate (IPP) under the catalysis of 1-deoxy-D-xylulose 5-phosphate synthase (DXS), 1-deoxy-D-xylulose 5-phosphate reductoisomerase (DXR), 2-C-methyl-D-erythritol 4-phosphate cytidylltransferase (MCT), 4-diphosphocytidyl-2-C-methyl-D-erythritol kinase (CMK), 2-C-methyl-Derythritol 2,4-cyclodiphosphate synthase (MDS), 1-hydroxy-2-methyl-2-(E)-butenyl 4-diphosphate synthase (HDS), and 1-hydroxy-2-methyl-2-(E)-butenyl 4-diphosphate reductase (HDR), respectively. The MVA pathway produces intermediates 3-hydroxy-3-methylglutaryl coenzyme A (HMG-CoA), mevalonate (MVA), mevalonate-5-phosphate (MVAP), mevalonate-5-Diphosphate (MVAPP), and isopentenyl diphosphate (IPP) under the catalysis of acetyl-CoA C-acetyltransferase (AACT), 3-hydroxy-3-methylglutaryl-CoA synthase (HMGS), 3-hydroxy-3-methylglutaryl-CoA reductase (HMGR), mevalonate kinase (MK), 5-phosphomevalonate kinase (PMK), and mevalonate pyrophosphate decarboxylase (MDC), respectively. IPP may convert to dimethylallyl diphosphate (DMAPP) under the catalysis of isopentenyl diphosphate isomerase (IDI). The formation of diphosphate precursor geranylgeranyl diphosphate (GGPP) is under the catalysis of geranylgeranyl diphosphate synthase (GGPPS). Red represents DMR-related genes. **b** Integrative Genomics Viewer display of DNA methylation levels of DMR-related enzyme genes.

### Regulation of tanshinone biosynthesis through mCHH at the promoter and the downstream regions of key enzyme genes

**Figure 7:**
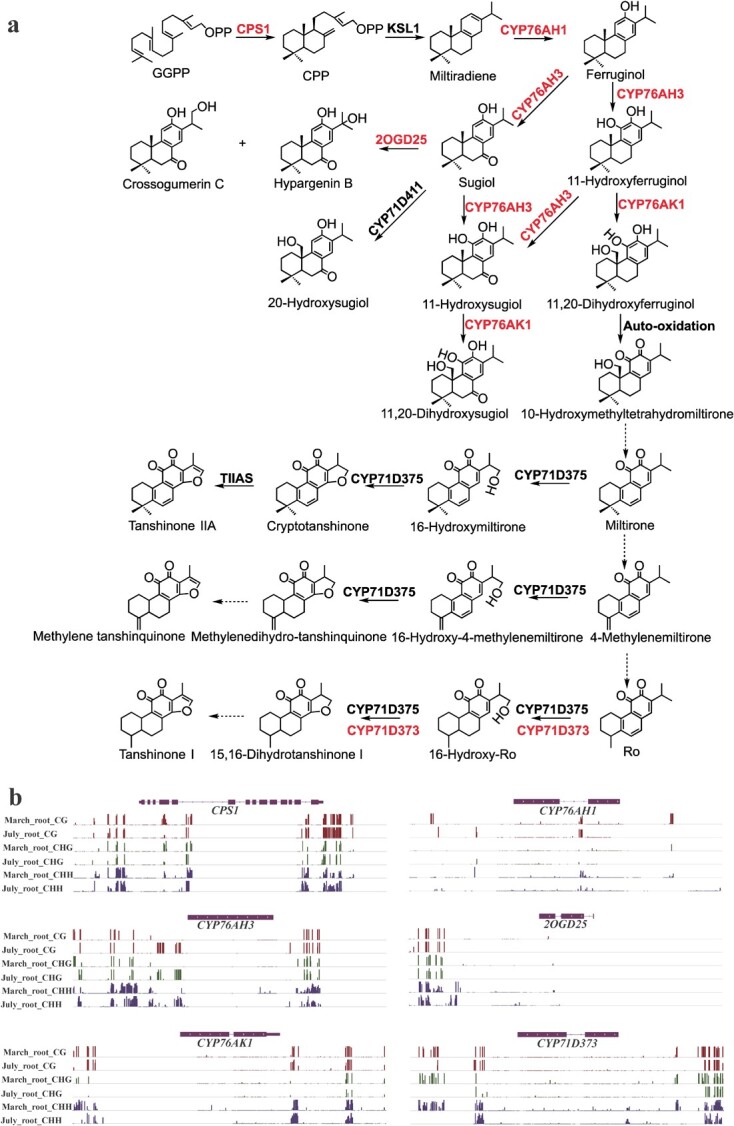
DMR-related enzyme genes in downstream pathway of tanshinone biosynthesis. **a** Tanshinone-related diterpenoid biosynthesis downstream pathway. Red represents DMR-related enzyme genes. Solid and dashed arrows indicate established and hypothetical relationships, respectively. **b** Integrative Genomics Viewer display of DNA methylation levels of DMR-related enzyme genes.

Tanshinones are a group of diterpenoids and the major lipophilic bioactive components in *S. miltiorrhiza* [40]. Its biosynthetic pathway involves at least 26 genes [34]. To further investigate its role in tanshinone biosynthesis, we comparatively analysed the methylation of tanshinone biosynthesis-related genes at three cytosine DNA sequence contexts. Among the 26 genes analyzed, four genes, including *HMGR1*, *DXR*, *CPS1*, and *CYP76AH3*, were hyperDMR-related; 11 genes, including *HMGR2*, *DXS2*, *DXR*, *CMK*, *MDS*, *IDI1*, *GGPPS1*, *CYP76AH1*, *2OGD25*, *CYP76AK1*, and *CYP71D373* were hypoDMR-related in July_root compared with March_root (Table S6, see online supplementary material). It implies the importance of DNA demethylation in tanshinone biosynthesis. For hyperDMR-related genes, *HMGR1* had promoter-related CHHDMR; *DXR* and *CPS1* both contained a CHHDMR overlapping with gene body and downstream; *CYP76AH3* included a DMR displaying all sequence context differential methylation and a DMR displaying symmetric sequence context differential methylation, which overlapped with gene promoter and body as well as body and downstream, respectively (Fig. 6b and 7b; Table S6, see online supplementary material). For hypoDMR-related genes, *CMK* contained a CHGDMR in gene body; *DXS2*, *CYP76AH1*, and *CYP76AK1* had a CGDMR overlapping with gene downstream, respectively; the remaining genes were all CHHDMR-related. Among CHHDMR-related genes, *DXS2*, *CMK*, and *IDI1* had CHHDMR-related promoters. Other genes, including *HMGR2*, *DXR*, *MDS*, *CYP76AH1*, *2OGD25*, and *CYP71D373*, had CHHDMR overlapping with gene downstream (Fig. 6b and 7b; Table S6, see online supplementary material). Further expression analysis showed that all tanshinone biosynthetic pathway genes displayed up-regulation in July_root compared to March_root (Fig. S9, see online supplementary material). These results suggest the key regulatory role of CHH DNA demethylation at the promoter and downstream regions in the biosynthesis of tanshinones. For DMRs in tanshinone biosynthetic pathway genes between July_root and March_root, although most DMRs were hypo-methylated in July_root, these DMRs had slightly higher 24-nt sRNA abundance in July_root (Table S6, see online supplementary material). There may be some complicated reasons for this phenomenon. For example, not all 24-nt sRNAs were involved in RdDM; some other sRNA species could contribute to DNA methylation of genes involved in tanshinone biosynthesis. A total of 11 DMR-related tanshinone biosynthetic pathway genes were identified between July_root and July_leaf. Among them, *HMGR1*, *HMGR2*, *DXR*, *MDS*, *HDR2*, *CPS1*, *KSL1*, *CYP76AH3*, and *CYP71D375* contained hyper-DMRs in July_root, whereas *PMK*, *DXR*, and *CMK* had hypo-DMRs (Table S7, Fig. S10, see online supplementary material). However, most genes showed higher expression in July_root (Fig. S11, see online supplementary material). It implies that DNA methylation is not the main mechanism in regulating the expression of tanshinone biosynthesis-related genes in leaves. Previous studies found that *Arabidopsis* employed distinctive epigenetic modifications (DNA methylation, repressive histone markers) to regulate distinct genomic loci in different tissues [52]. A similar situation could exist for *S. miltiorrhiza* July_leaf.

**Figure 8:**
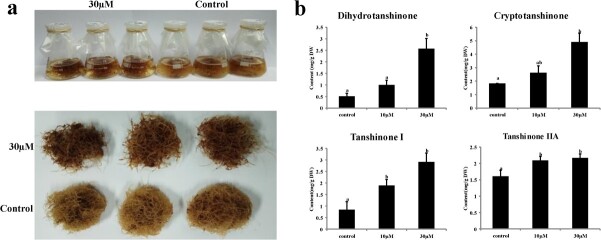
The effects of 5-azacytidine on tanshinone biosynthesis in the hairy roots of *S. miltiorrhiza*. **a** The phenotype of hairy roots with or without 5-azacytidine treatment. **b** The contents of dihydrotanshinone I, cryptotanshinone, tanshinone I, and tanshinone IIA in the hairy roots of *S. miltiorrhiza* treated with different levels of 5-azacytidine for 30 days. Average values from three biological replicates are shown. The error bars represent SE. Significant differences at the 5% level calculated through one-way ANOVA analysis are indicated by different letters.

To detect the significance of DNA demethylation in tanshinone biosynthesis, a DNA methylation inhibitor, 5-azacytidine, was applied to the hairy roots of *S. miltiorrhiza*. The results showed that the hairy roots with 5-azacytidine treatment were much redder than those without treatment (Fig. 8a). Further measurement of cryptotanshinone, tanshinone I, dihydrotanshinone, and tanshinone IIA using ultra-performance liquid chromatography (UPLC) showed that the contents of four tanshinones were significantly increased after 5-azacytidine treatment, particularly in those hairy roots treated with 30 μM 5-azacytidine (Fig. 8b). Control and 30 μM 5-azacytidine-treated *S. miltiorrhiza* hairy roots were subjected to whole genome bisulfite sequencing. DNA methylation and expression levels of tanshinone biosynthetic pathway genes were analysed. The results showed that almost all genes were down-regulated in DNA methylation levels and up-regulated in expression levels under 5-azacytidine treatment (Figs S12 and S13, see online supplementary material). Further DMR analysis showed that all of the identified DMR-related genes contained hypoDMRs in 30 μM 5-azacytidine-treated *S. miltiorrhiza* hairy roots except *CYP76AK1* (Fig. S14, Table S8, see online supplementary material). Taken together, these results showed that DNA methylation inhibitor treatment could promote tanshinone production through demethylation of tanshinone biosynthetic pathway genes in *S. miltiorrhiza* hairy roots.

## Discussion

Medicinal plants are usually rich in bioactive compounds, which are the key ingredients of traditional medicines and important sources of chemical medicine [53]. In addition, they are also used in food supplements, food flavoring agents, perfumes, and cosmetics. Therefore, the biosynthesis and regulatory mechanism of bioactive compounds is always a main topic in medicinal plant studies. Recently, significant progress has been made in the identification of genes encoding key enzymes of the biosynthetic pathways, transcriptional factors regulating active component biosynthesis, and non-coding RNAs regulating bioactive compound production [34]. However, the methylome of medicinal plants and the regulatory mechanism of DNA methylation in bioactive compound biosynthesis are largely unknown. This study is the first effort in characterizing the methylome of *S. miltiorrhiza*, a model system for medicinal plant biology [34].

Comparing with the model plant *Arabidopsis* and other plant species, such as tea and orange [54, 55], we found that the DNA methylation pathways could be deeply conserved in *S. miltiorrhiza*, because the homologs of all known genes associated with the pathways in other plants exist in the *S. miltiorrhiza* genome (Table 1) [44, 56–58]. Consistently, *S. miltiorrhiza* and other plants share many methylome characteristics, such as the positive correlation between the level of DNA methylation and the coverage of TEs, the negative correlation between the level of DNA methylation and the density of genes, the higher level of DNA methylation in TEs than genic regions, the increase of mCG in gene bodies and the flanking regions and its reduction in transcriptional start/end sites, the increase of mCHG and mCHH in the flanking regions and their reduction in gene bodies, and the higher level of DNA methylation in TE bodies than the upstream and downstream regions (Fig. 1) [16–19]. In addition, higher degrees of DNA methylation in lowly expressed genes and TEs with 24-nucleotide sRNA distribution were also commonly found in plants (Figs 2 and 3) [20, 54].

The regulatory role of DNA methylation in gene expression has been widely analysed [16, 26]. Generally speaking, methylation at gene promoters will inactivate or reduce gene transcription, and methylation at coding regions will inhibit transcript elongation [3, 6, 8]. However, in *S. miltiorrhiza*, the relationship between DNA methylation and gene expression is complicated. There is no reverse correlation between gene body mCG and gene expression (Fig. 2a). The heaviest gene body mCG was observed in moderately highly expressed genes (10 < FPKM ≤50), and the relatively low gene body mCG showed relatively low expression (0 < FPKM ≤1) (Fig. 2b). The underlying mechanism remains to be elucidated.

Comparative analysis of DNA methylation in three *S. miltiorrhiza* samples (March_root, July_root, and July_leaf) reveals the significance of mCHH in regulating gene expression. The significance is evidenced not only by the differences of average level of DNA methylation between March_root and July_root and between July_leaf and July_root, but also by the number of hypoDMC/DMRs and hyperDMC/DMRs among *S. miltiorrhiza* samples. A significant contribution of mCHH in regulating gene expression was also found in other plants, such as *Prunus mume* [31] and mungbean [59]. It suggests the sensitivity of mCHH during plant development and in response to environments [60, 61].

Regulation of plant bioactive compound biosynthesis is complicated. The regulatory networks are usually multilayered, involving abiotic and biotic factors, plant hormones, and growth regulators, DNA methylation, noncoding RNAs, transcription factors, and so on [34]. Among them, DNA methylation is an important epigenetic mark, which connects external factors and internal gene expression. Involvement of DNA methylation in secondary metabolism has been found in the biosynthesis of linalool and inonone [32], carotenoids [62], anthocyanins [33], and floral volatiles [31]. Tanshinones are a group of diterpenoid compounds. Their biosynthetic pathway can be divided into four stages. They include biosynthesis of C_5_ isoprene units through the MEP and the MVA pathways, formation of diphosphate precursor GGPP, formation of parent carbon skeleton miltiradiene, and production of final tanshinone products [34]. Comparative analysis of DNA methylation in July_root and March_root of *S. miltiorrhiza* showed that 14 of the 26 tanshinone biosynthesis enzyme genes analysed were DMR-related. Analysing the location of DMRs showed that, among the 14 DMR-related genes, three (*HMGR1*, *IDI1*, and *GGPPS1*) had DMRs in the promoter region, six (*MDS*, *HMGR2*, *CYP76AH1*, *CYP76AK1*, *CYP71D373*, and *2OGD25*) had DMRs in the 3′ downstream region, and the other five (*DXS2*, *DXR*, *CMK*, *CPS1*, and *CYP76AH3*) had DMRs in at least two regions of promoter, gene body and 3′ downstream. The most significant changes were found for mCHH demethylation in the promoters and the 3′ flanking regions. It accounts for 50% of total DMRs identified for three sequence contexts in promoters, gene bodies, and 3′ downstream regions. GO analysis showed that genes associated with diterpenoid biosynthesis were significantly enriched in those with downstream overlapping with hypoCHHDMRs and DNA inhibitor promoted tanshintone biosynthesis in hairy roots (Figs 5 and 8). It suggests the significance of DNA methylation in regulating tanshinone biosynthesis, particularly mCHH demethylation in the promoter and the downstream regions.

Among the 14 DMR-related genes, seven are involved in the biosynthesis of C_5_ isoprene units. *DXS2*, which encodes 1-deoxy-D-xylulose 5-phosphate synthase, is the first enzyme of the MEP pathway. It catalyzes the production of 1-deoxy-D-xylulose 5-phosphate (DXP) through the condensation of glyceraldehyde 3-phosphate (G3P) and pyruvate [40]. Overexpression of *DXS2* significantly enhanced tanshinone accumulation in *S. miltiorrhiza* and increased chlorophyll a and gibberellin contents in *Arabidopsis* [63]. *DXR* encodes 1-deoxy-D-xylulose 5-phosphate reductoisomerase. It is the second enzyme of the MEP pathway. DXR catalyzes the rearrangement and reduction of DXP [40]. Overexpession of *DXR* promoted the accumulation of tanshinone in *S. miltiorrhiza* and the production of β-carotene in *Escherichia coli* [64]. Different from *DXS2* and *DXR* that are involved in the MEP pathway, *HMGR1* and *HMGR2* are the key enzyme genes involved in the MVA pathway [40]. Overexpression of *HMGR1* and *HMGR2* enhanced tanshinone and squalene production in *S. miltiorrhiza* [64–66]. In addition, *IDI1* encodes isopentenyl diphosphate isomerase. It catalyzes the reversible conversion of isopentenyl diphosphate (IPP) and its isomer 1-hydroxy-2-methyl-2-(E)-butenyl 4-diphosphate (HMBPP) [40]. Down-regulation of *IDI1* expression through RNA interference approach caused significant reduction of tanshinone production in *S. miltiorrhiza*, whereas its expression in *E. coli* promoted carotenoid biosynthesis [67]. The changes of DNA methylation in these genes in July_root and March_root indicate that DNA methylation could be involved not only in tanshinone biosynthesis, but also in the production of other terpenoids.

*GGPPS1* encodes the key enzyme associated with the second stage of tanshinone biosynthesis [40]. It catalyzes IPP and DMAPP condensation to form the intermediate diphosphate precursor GGPP for diterpenoids, carotenoids, chlorophylls, and tetrapenes. Overexpression of *GGPPS1* could significantly enhance the biosynthesis of tanshinones in *S. miltiorrhiza* [66]. Changes of DNA methylation in *SmGGPPS1* promoter could be important for the biosynthesis of diterpenoids and its derivatives. The other DMR-related genes, including *CPS1*, *CYP76AH1*, *CYP76AH3*, *CYP76AK1*, *CYP71D373*, and *2OGD25*, are more specific to tanshinone biosynthesis compared with those discussed above. Among them, *CPS1* encodes copalyl diphosphate synthase catalyzing the cyclization of GGPP to copalyl diphosphate (CPP) [68, 69]. *CYP76AH1*, *CYP76AH3,* and *CYP76AK1* catalyze successive reaction steps that convert miltiradiene to 11,20-dihydroxy sugiol and 11,20-dihydroxy ferruginol [41, 42], *CYP71D373* is involved in 15,16-dihydrotanshinone I biosynthesis [38], whereas *2OGD25* is involved in the hydroxylation of sugiol at C-15,16 [70]. The changes of DNA methylation in these key enzyme genes were correlated with their expression in roots of *S. miltiorrhiza* line 99–3 collected in March and July (Fig. S9, see online supplementary material). It is also consistent with the results from DNA methylation inhibitor treatment (Fig. 8). These data provide solid evidence for the regulatory role of DNA methylation in tanshinone biosynthesis.

## Conclusions

We have previously shown that DNA methylation could be involved in regulating bioactive compound biosynthesis in *S. miltiorrhiza* [44, 45]. However, it is unknown how global DNA methylation change may contribute to tanshinone biosynthesis. This study provides the first genome-wide DNA methylation landscape at single base resolution in *S. miltiorrhiza*. The results showed that low DNA methylation could up-regulate the expression of genes and 24 nt sRNA could be the main participant for RdDM pathway in *S. miltiorrhiza*. In addition, DMC/DMR analysis showed that differential methylation predominantly occurred in CHH sequence context and genes related to hypoCHHDMR in July_root were enriched in terpene biosynthesis process in comparison with those in March_root. Most importantly, among the 14 DMR-related tanshinone biosynthesis enzyme genes between July_root and March_root, nine, including *DXS2*, *CMK*, *IDI1*, *HMGR2*, *DXR*, *MDS*, *CYP76AH1*, *2OGD25*, and *CYP71D373*, displayed lower CHH methylation in gene promoter or downstream regions in July_root. Further DNA methylation inhibitor treatment promoted tanshinone biosynthesis in *S. miltiorrhiza* hairy roots. Taken together, DNA methylation could promote tanshinone biosynthesis through CHH demethylation in promoter and downstream of tanshinone biosynthesis enzyme genes. This research provides novel insight into the epigenetic regulatory mechanism of tanshinone biosynthesis and will be helpful for further improvement of active compound production in *S. miltiorrhiza*.

## Materials and methods

### Plant materials and growth conditions

*S. miltiorrhiza* line 99–3 plants were grown under natural conditions in an experimental field at the Institute of Medicinal Plant Development (IMPLAD). March_root samples were collected from two-year-old plants in late March. July_root and July_leaf samples were collected in late July. The harvested plant materials were immediately frozen in liquid nitrogen until use.

### BS-seq library construction and genome bisulfite sequencing

Genomic DNA was isolated from *S. miltiorrhiza* samples using the QIAamp DNA Mini Kit (Qiagen, Valencia, CA, USA). NanoPhotometer® spectrophotometer (IMPLEN, CA, USA) and agarose gel electrophoresis were employed to monitor the integrity and purity of isolated genomic DNA. For each sample, Covaris S220 was used to fragment 5.2 microgram genomic DNA spiked with 26 ng lambda DNA to 200-300 bp. The fragmented DNA was end-repaired and adenosinated. Methylated cytosine barcodes were ligated to the fragmented genomic DNA following the manufacturer’s protocols. Then, the EZ DNA Methylation-Gold TM kit (Zymo Research, CA, USA) was used to bisulfate-convert DNA fragments twice. After PCR amplification, library concentration was assayed by the Qubit® 2.0 Flurometer (LifeTechnologies, CA, USA). The size of insertion was assayed by Agilent Bioanalyzer 2100 system. An Illumina Hiseq 2000 platform was used to sequence the qualified libraries.

### Bisulfite sequencing data alignment and methylation level calculation

Raw WGBS reads were processed to filter adapters and low-quality reads using the Trimmomatic software [71]. The obtained clean reads were aligned to whole genome of *S. miltiorrhiza* line 99–3 using Bismark v0.23.0 under the following paramaters: —bowtie2 -q —maxins 500 –directional —score-min L, 0, −0.2 —no-discordant —ignore-quals –dovetail —no-mixed [72]. The Bismark methylation extractor was used to call methylated cytosines from uniquely mapped reads. Cytosines with at least four reads were estimated for methylation level, which was calculated through dividing the number of Cs by the number of Cs plus Ts. Lambda DNA was used to calculate bisulfite conversion rate. Methylation level of genes and TEs was analysed through proportionally dividing the upstream and downstream 2 kb regions and the body regions into 20 bins, respectively. All genes and TEs were used to calculate average methylation level for each bin.

### Differential methylation analysis

DMC and DMR were identified using Fisher’s exact test and Benjamini–Hochberg FDR. The cytosine was defined as DMC with absolute methylation difference of 0.2 and FDR < 0.05. The genome of *S. miltiorrhiza* was divided into 1000 bp bins and methylation levels were compared. The bin was considered as DMR with FDR <0.01 and the difference of absolute methylation level of 0.2, 0.15, and 0.1 for CG, CHG, and CHH context, respectively.

### RNA sequencing analysis and qRT-PCR

TRIzol reagent (Ambion, Austin, TX, USA) was used to isolate RNA from plant tissues. Two percent agarose gels and NanoPhotometer spectrophotometer (IMPLEN, CA, USA) were used to monitor the integrity and purity of RNA. RNA concentration was estimated using the Qubit RNA Assay kit and Qubit 2.0 Flurometer (Life Technologies, CA, USA). The qScript cDNA SuperMix kit (Quanta, Gaithersburg, MD, USA) was used for conversion of one μg of RNA to cDNA. Sequencing library of March_root was generated using the NEBNext Ultra™ RNA Library Prep kit (NEB, MA, USA). Illumina Hiseq 2000 platform was used to sequence the library for 150-bp paired-end reads. Trimmomatic was used to remove adapters and low-quality reads [70]. Clean reads were mapped to the genome assembly of *S. miltiorrhiza* line 99–3 using Hisat2 [73]. Gene expression values were calculated as FPKM (Fragments Per Kilobase per Million) using Stringtie [73]. Real-time qRT-PCR was performed using the SYBR premix Ex Taq™ kit (TaKaRa, China). The results were detected using the CFX96™ real-time PCR detection system (Bio-Rad, USA). Each tissue was carried out in triplicate. *UBQ10* was an internal control. Relative expression of genes was analysed calculated using the 2^−ΔΔCt^ method. The primers are provided in Table S9 (see online supplementary material).

### sRNA sequencing and analysis

Isolation of total RNA from March_root and July_root was carried out using the TRIzol reagent (Ambion, Austin, TX, USA). sRNA library was constructed as per the Illumina TruSeq Small RNA Sample Preparation protocol and then sequenced on Illumina Hiseq 2000 platform for 50-bp single-end reads. Low-quality reads and adapters were removed from raw reads using Trimmomatic [71]. All clean reads were mapped to the genome of *S. miltiorrhiza* line 99–3 using Bowtie under the default parameters and allowing no mismatch [74]. The reads with unique alignment were used for further analysis.

### 5-Azacytidine treatment and tanshinone analysis

The 5Aza-dC dissolved in ddH_2_O was filtered by aseptic filter and diluted into two different concentrations (10 μM and 30 μM) in 6,7-V medium. A total of 0.1 g fresh hairy roots of *S. miltiorrhiza* were cultivated for 30 days in 6,7-V medium containing different 5Aza-dC concentrations (0, 10, and 30 μM). Then, the roots were harvested and dried to a constant weight under 45°C. One-gram dried samples were ground into powder and extracted for 40 min using ultrasonic bath. The obtained extracts were filtered for UPLC analysis using 0.2 μm organic membrane. Chromatography analysis was carried out using the Waters ACQUITY UPLC BCH C18 column with a mobile phase of 75% methanol (Waters, USA). The temperature was set at 30°C. The flow rate was set at 1 mL min^−1^.

## Acknowledgements

This work was supported by the CAMS Innovation Fund for Medical Sciences (CIFMS) (2021-I2M-2-001).

## Author contributions

S.L. and J.L. conceived and designed the experiments. J.L., C.L., and Y.D. performed the experiments. J.L. and H.W analysed the data. J.L. and S.L. wrote the manuscript. All authors read and agreed to the final article.

## Data availability

All sequencing data generated are available at the NCBI SRA database under accession number PRJNA975272.

## Conflict of interest statement

The authors declare no conflicts of interest.

## Supplementary data

Supplementary data is available at *Horticulture Research* online.

## Supplementary Material

Web_Material_uhad114
