# Supplementary material for: Characteristics of Salvia miltiorrhiza methylome and the regulatory mechanism of DNA methylation in tanshinone biosynthesis
Source: Hortic Res. 2023 May 31;10(7):uhad114. doi: 10.1093/hr/uhad114 (PMC10419789; doi:10.1093/hr/uhad114)
Supplement: Web_Material_uhad114 [file web_material_uhad114.zip › Supplemental files.docx]

**Supplemental files**


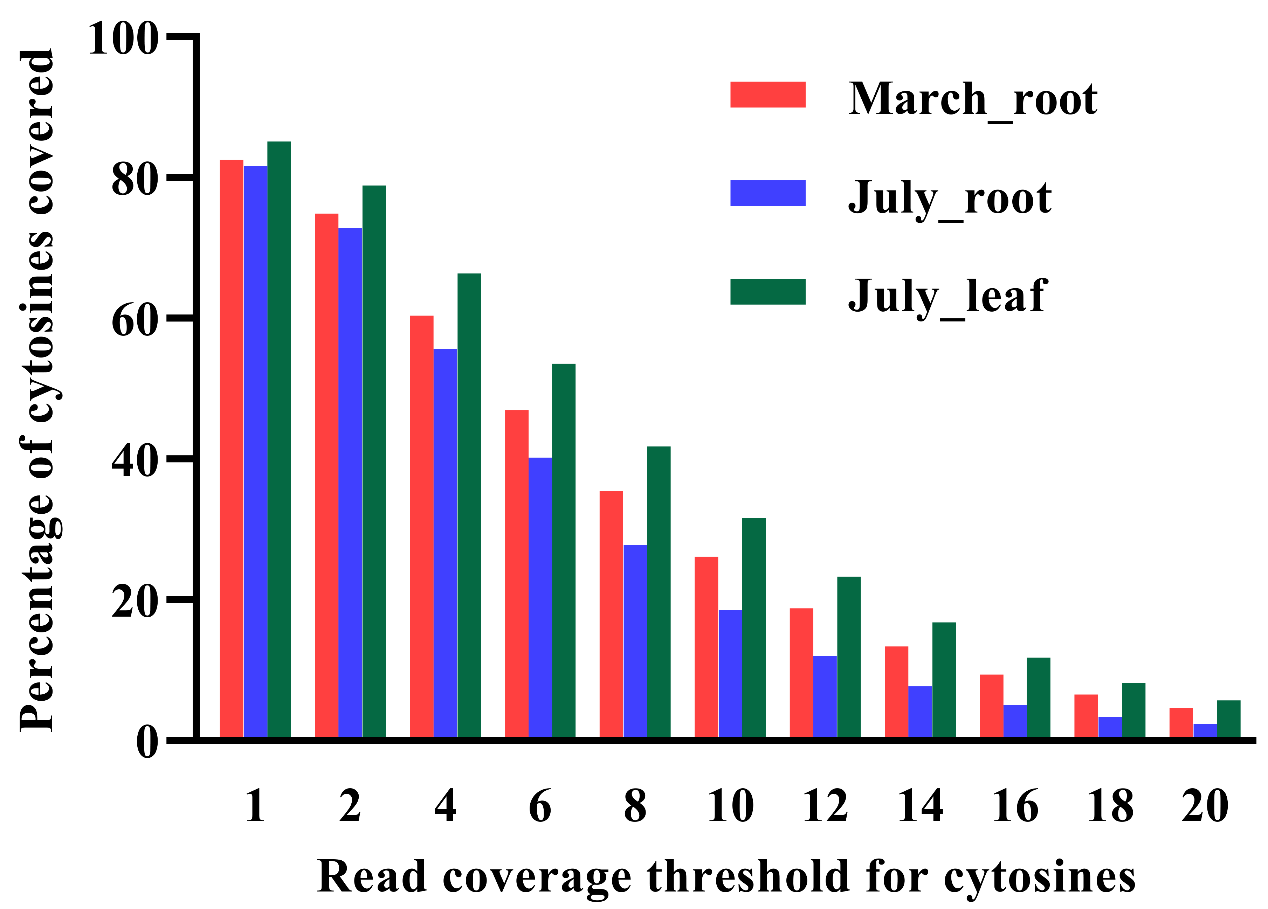


**Fig. S1. Cytosine proportion covered by at least BS-seq ‘X’ reads.** For example, about 80% of cytosines were covered by at least one read in all three samples.


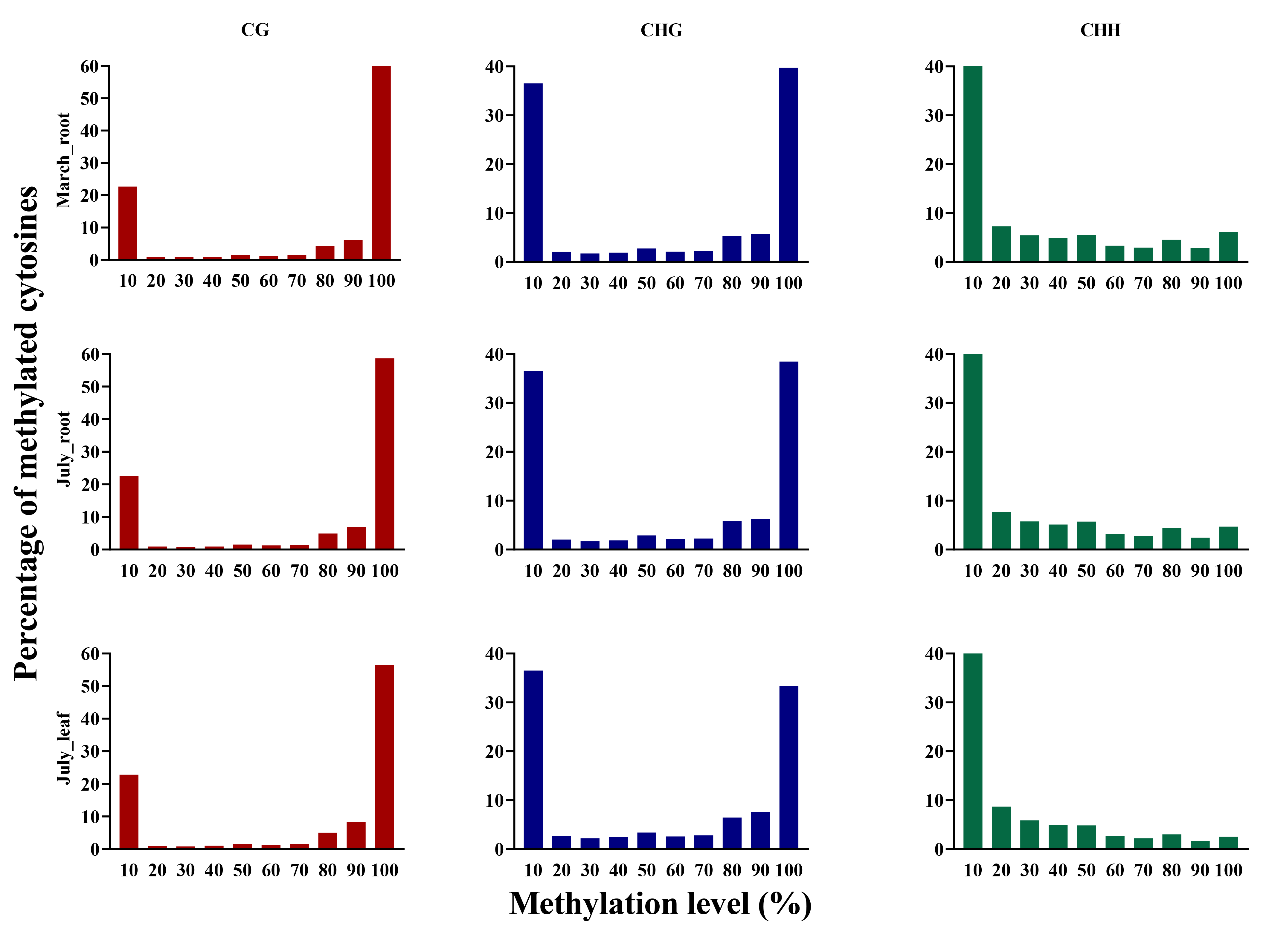


**Fig. S2. DNA methylation level distribution in each sequence context in three samples.** The methylation levels were divided into 10 bins from 10% to 100%.


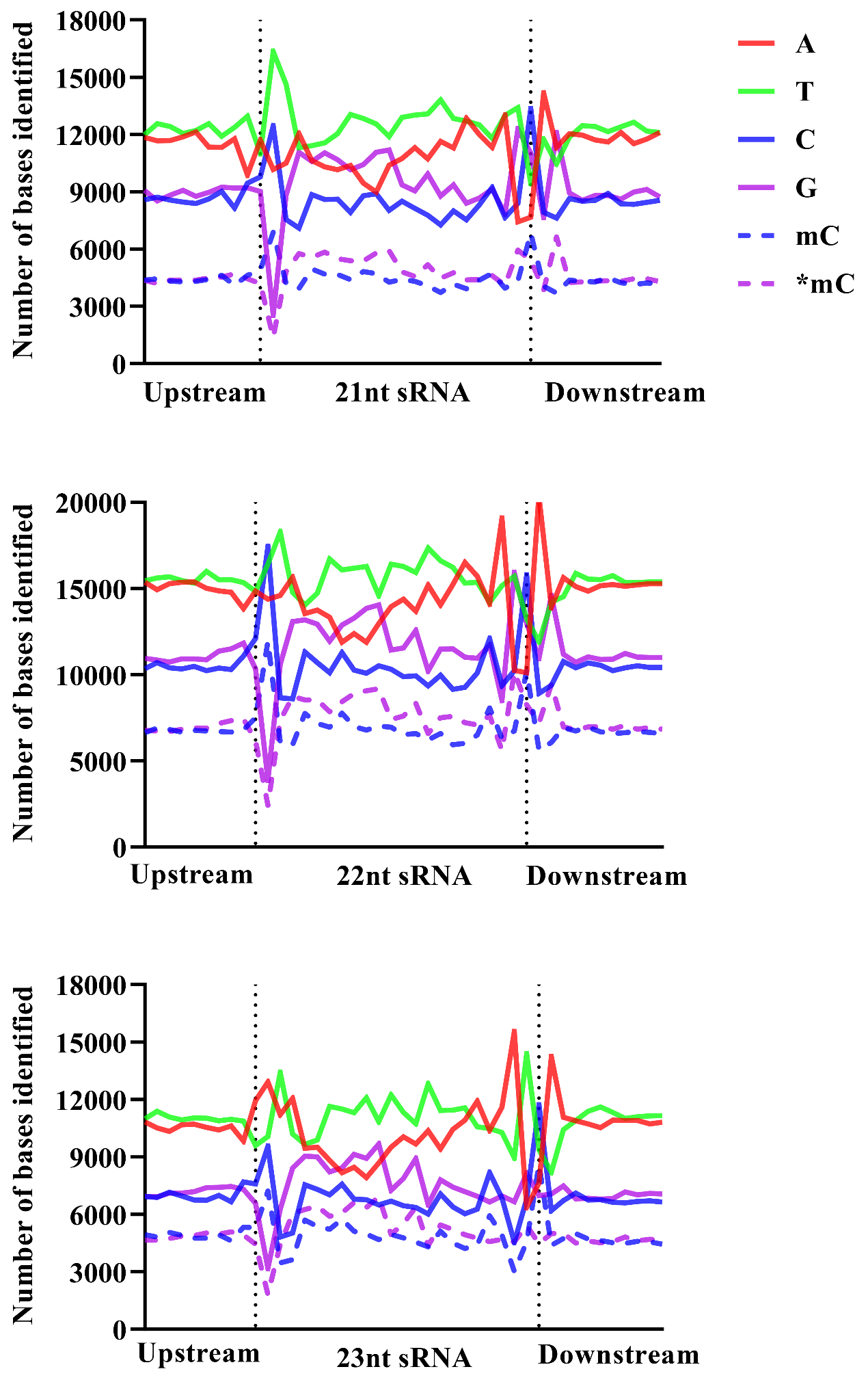


**Fig. S3. Relationship between 21-, 22-, 23-nucletide sRNAs and methylated cytosines.**

**
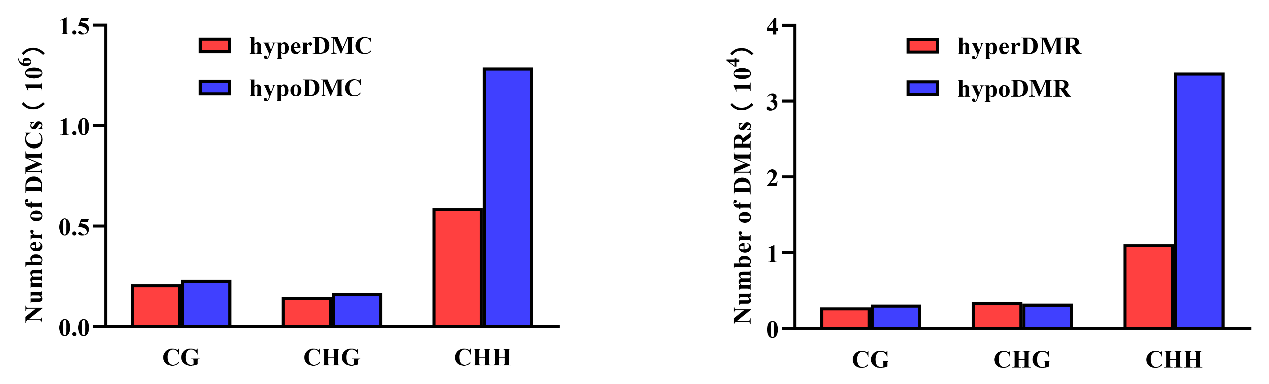
**

**Fig. S4. Numbers of DMCs and DMRs of each sequence context in July_root compared with March_root.**

**
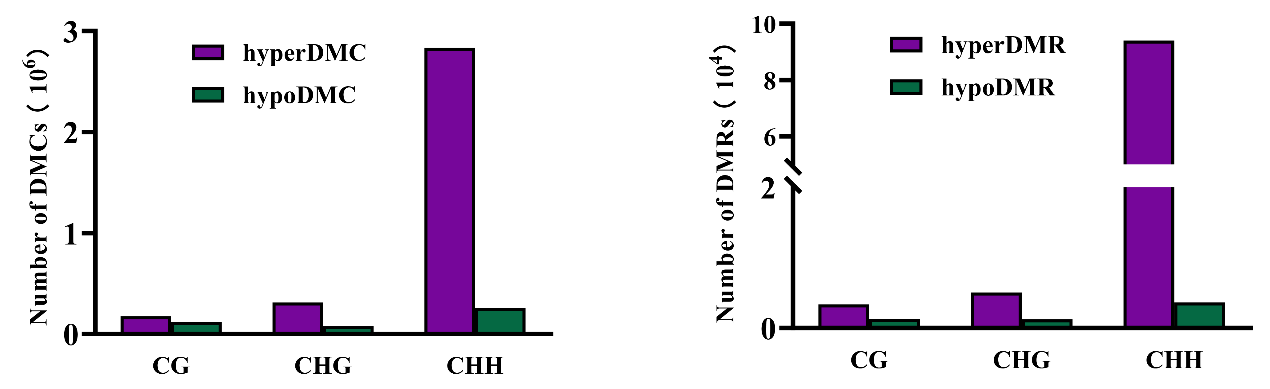
**

**Fig. S5. Numbers of DMCs and DMRs of each sequence context in July_root compared with July_leaf.**

**
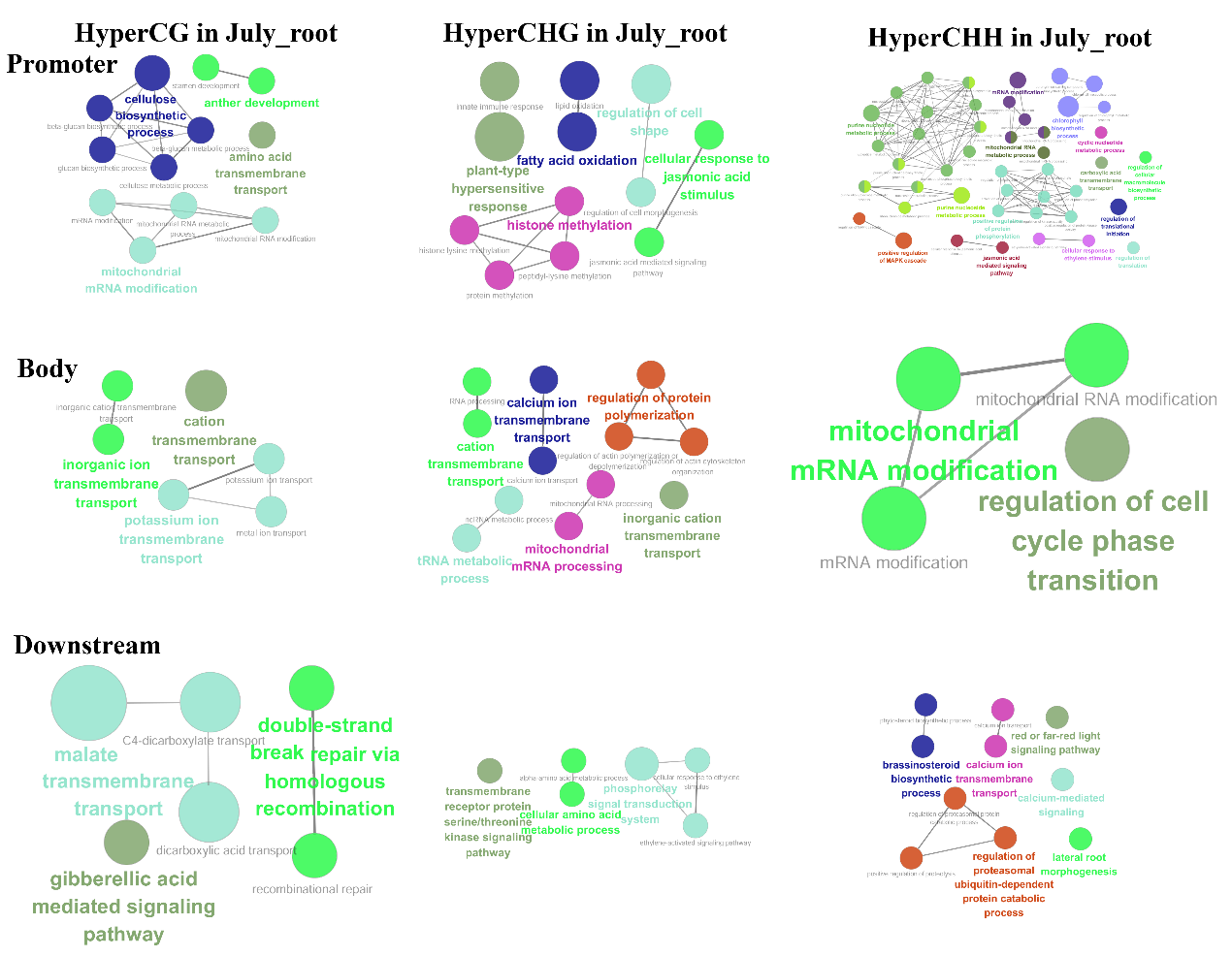
**

**Fig. S6. Biological processes significantly enriched in hyperDMR-related genes in July_root compared with March_root.**

**
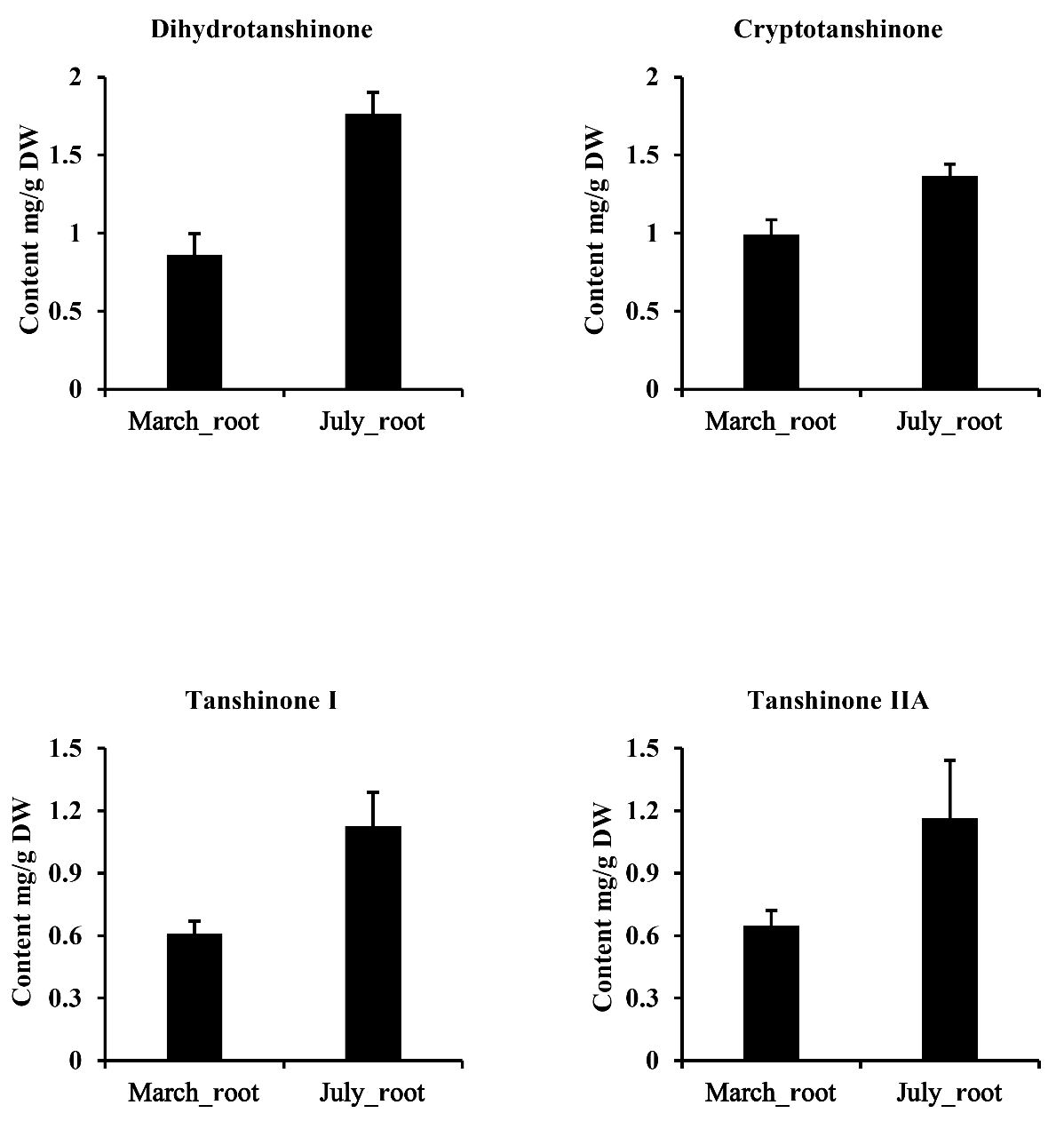
**

**Fig. S7. The contents of dihydrotanshinone I, tanshinone I, cryptotanshinone and tanshinone IIA in March_root and July_root.** Average values from three biological replicates are shown. The error bars represent SE.


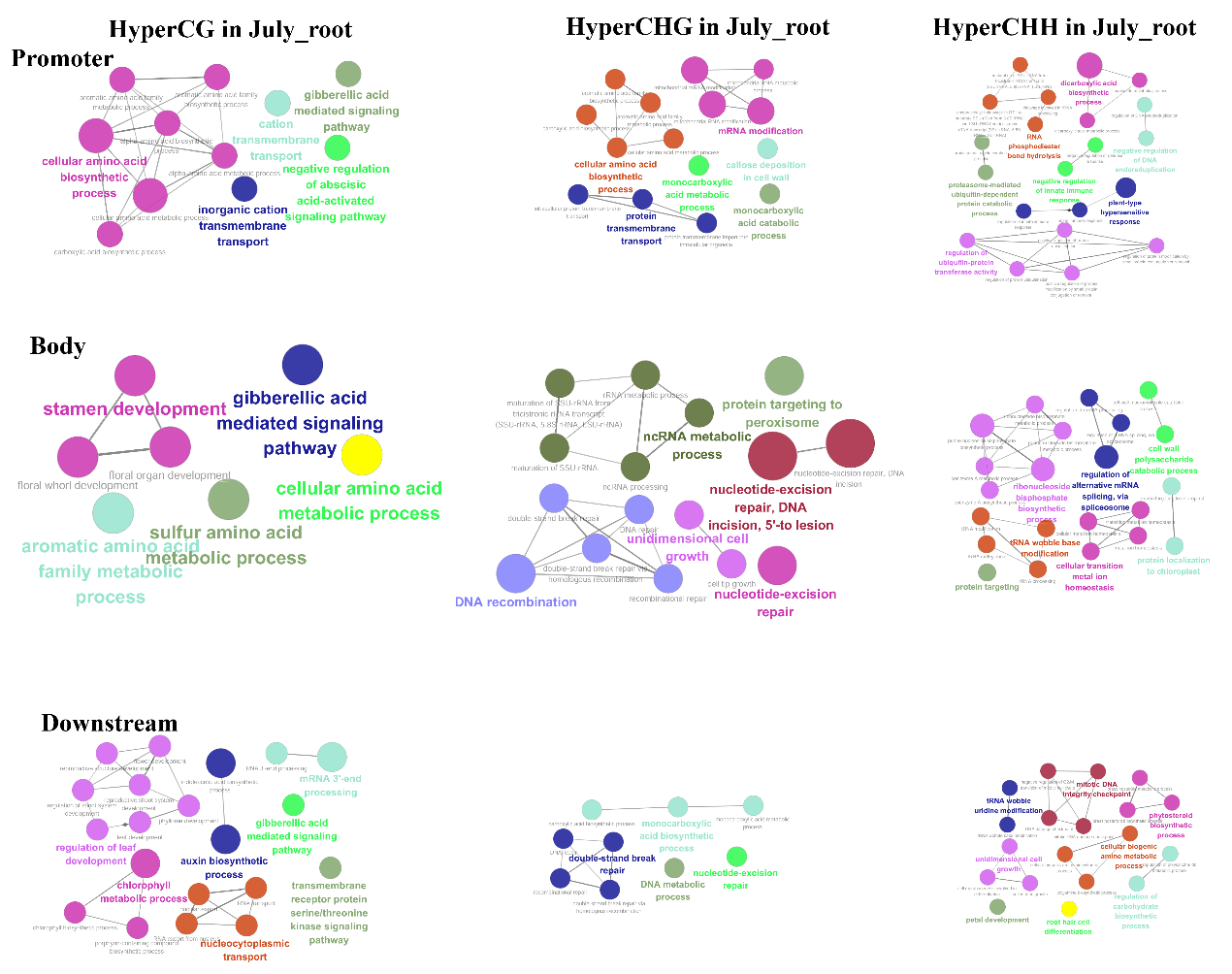


**Fig. S8. Biological processes significantly enriched in hyperDMR-related genes in July_root compared with July_leaf.**

**
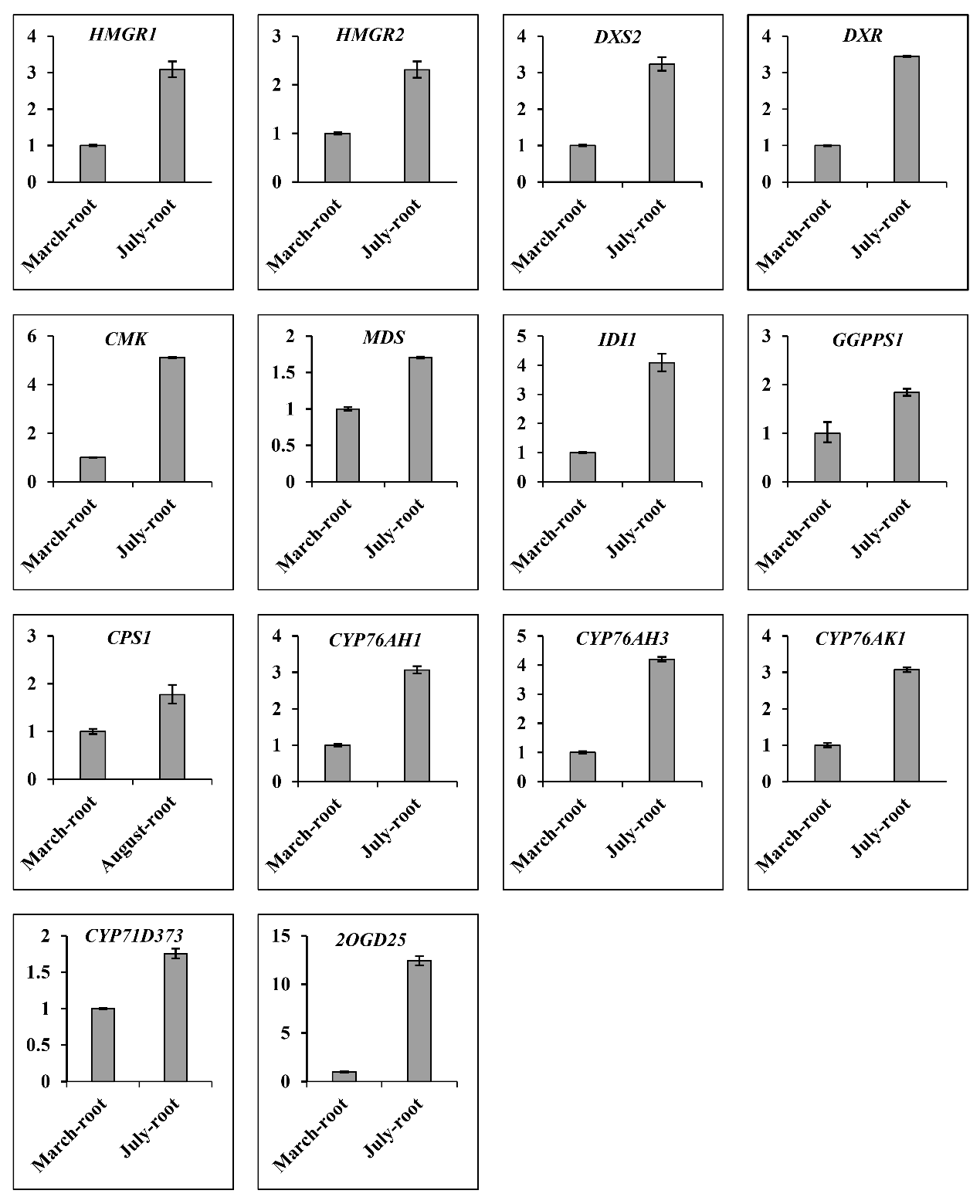
**

**Fig. S9. Expression patterns of DMR-related tanshinone biosynthesis enzyme genes in *S. miltiorrhiza* (99-3) root samples collected in March and July.** Transcript levels in March_root were arbitrarily set to 1.


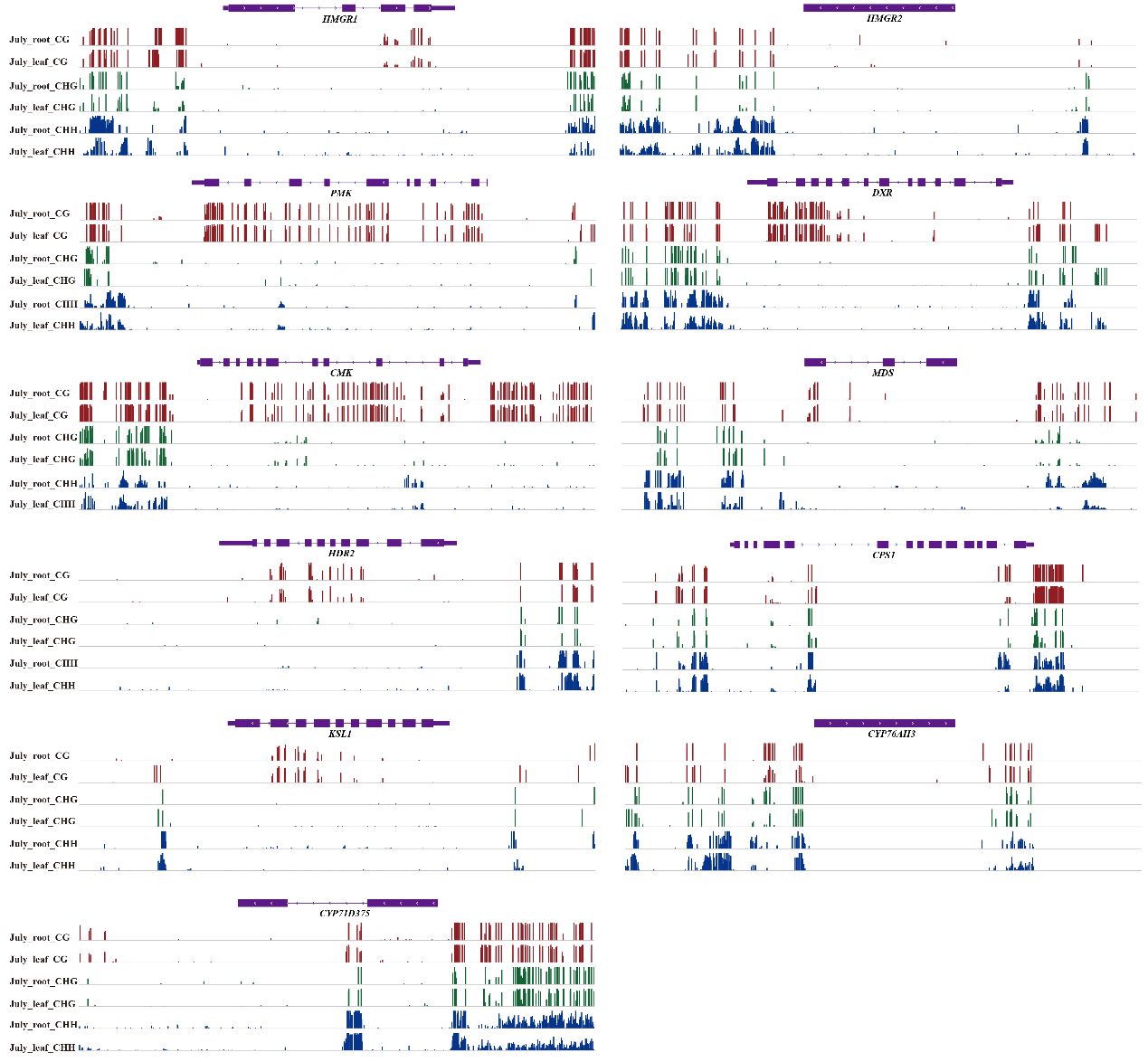


**Fig. S10. Integrative Genomics Viewer display of DNA methylation levels of DMR-related tanshinone biosynthesis enzyme genes between July_root and July_leaf.**

**
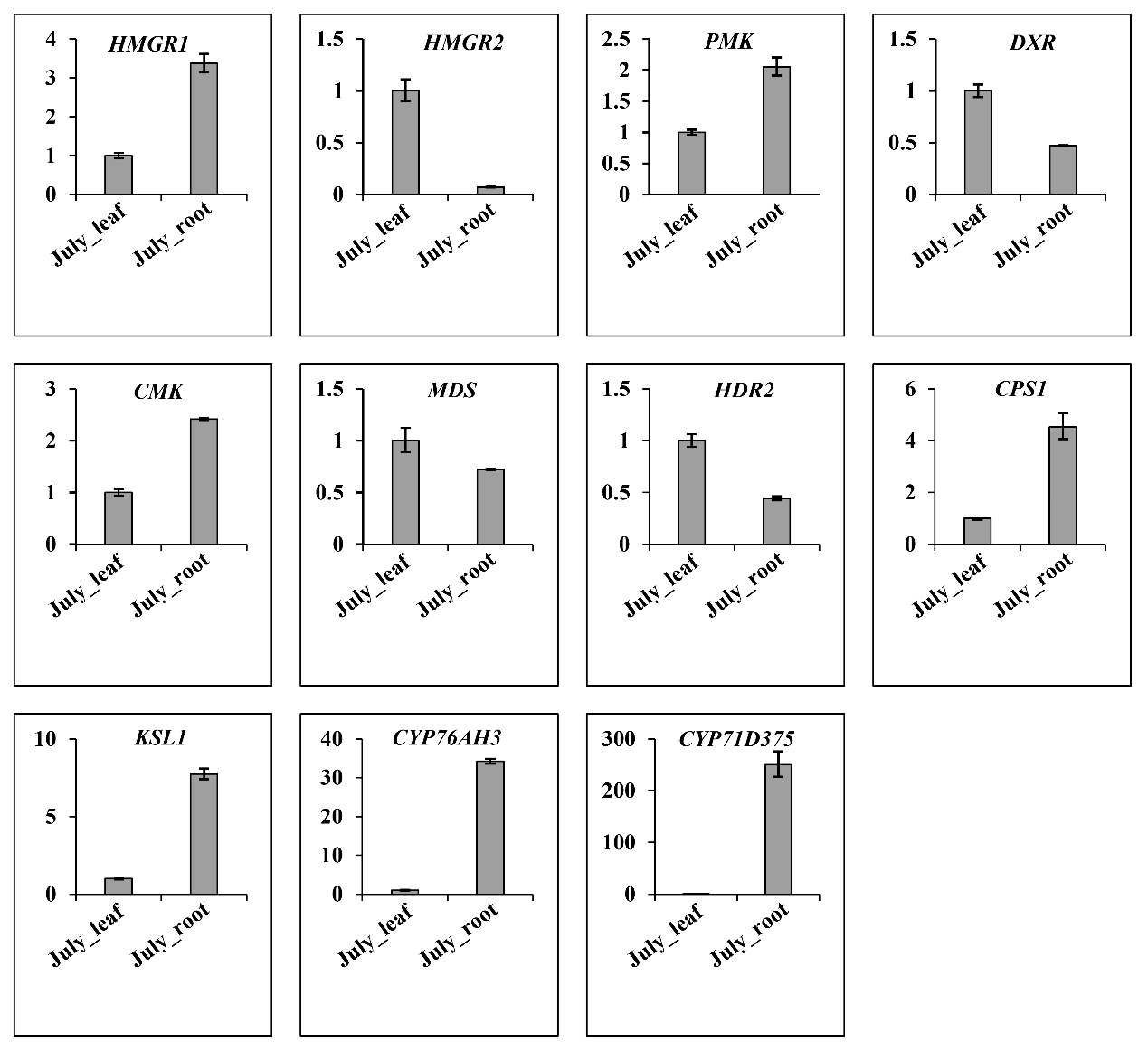
**

**Fig. S11. Expression patterns of DMR-related tanshinone biosynthesis enzyme genes in July_leaf and July_root.** Transcript levels in July_leaf were arbitrarily set to 1.


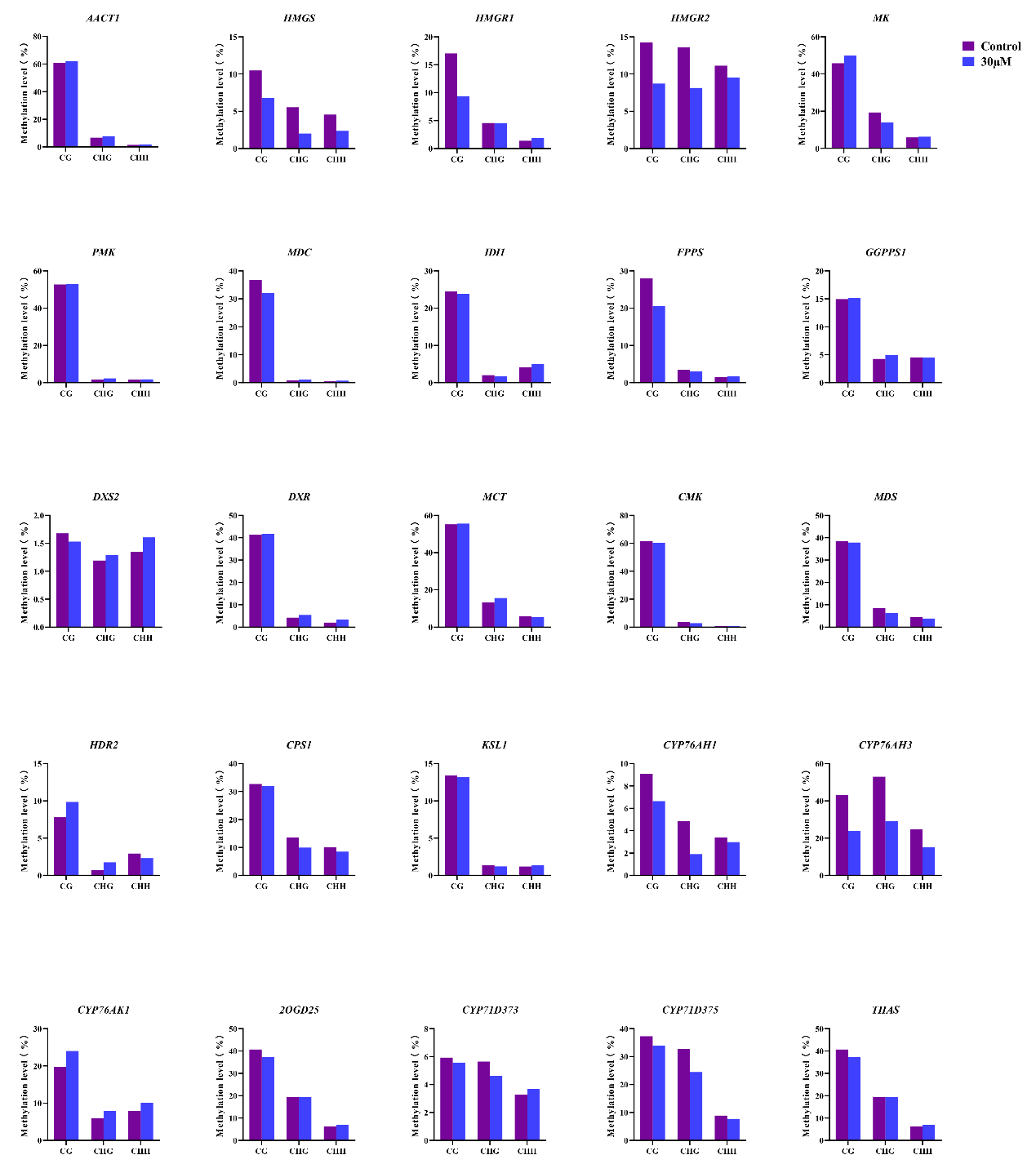


**Fig. S12. The effects of 5-azacytidine on the DNA methylation of tanshinone biosynthesis related genes in *S. miltiorrhiza* hairy roots.** Average methylation level was calculated from 2 kb region upstream to 2 kb region downstream of gene.


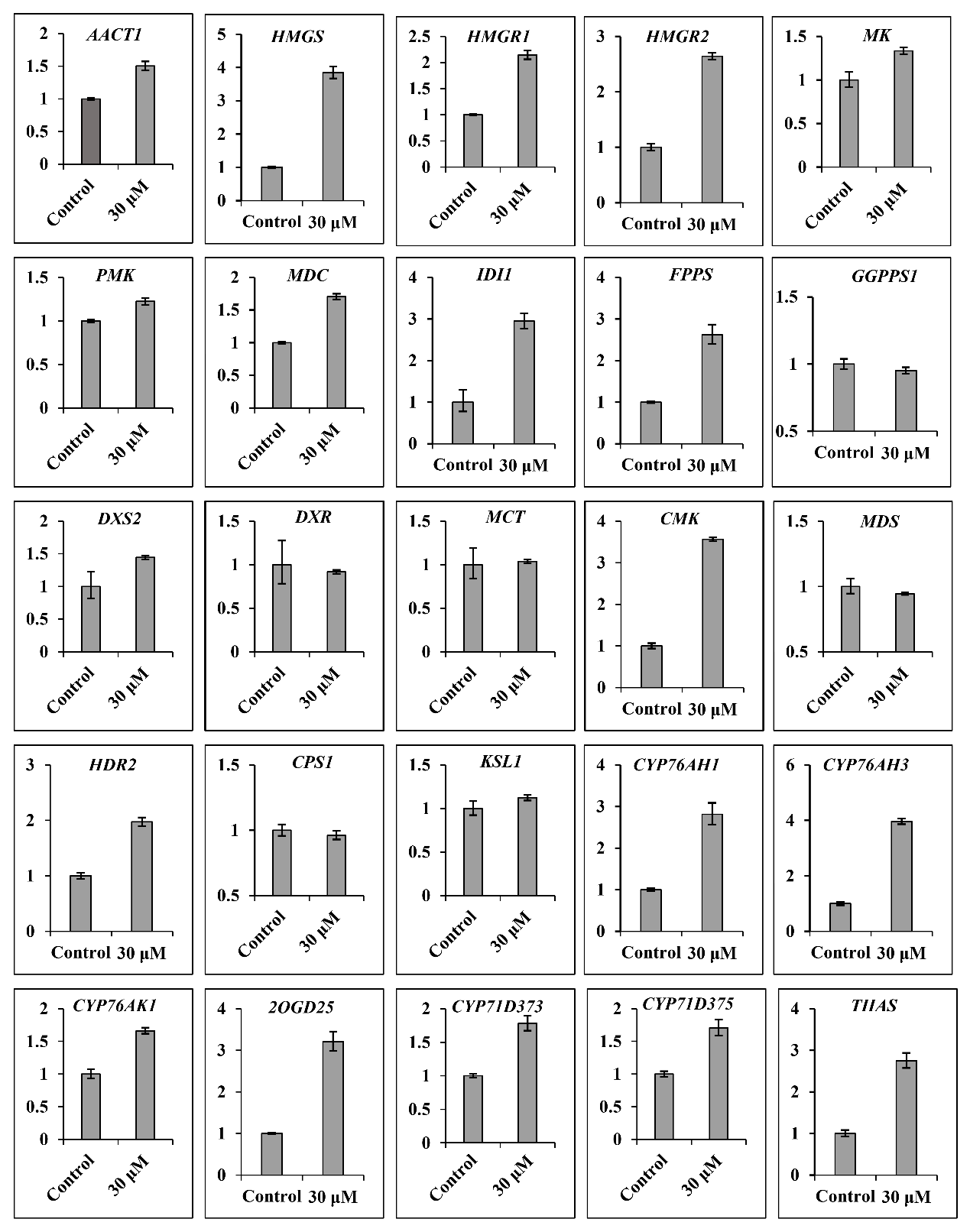


**Fig. S13. The effects of 5-azacytidine on the expression of tanshinone biosynthesis related genes in *S. miltiorrhiza* hairy roots.** Transcript levels in control were arbitrarily set to 1.


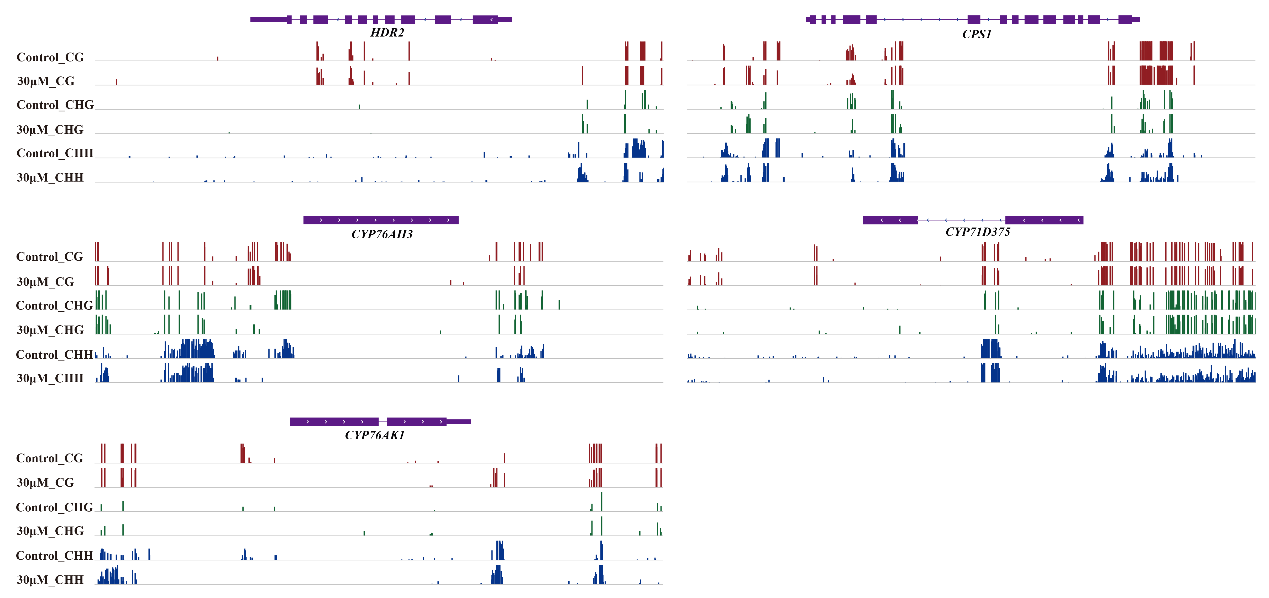


**Fig. S14. Integrative Genomics Viewer display of DNA methylation levels of DMR-related tanshinone biosynthesis enzyme genes between control and 30μM 5-azacytidine-treated *S.miltiorrhiza* hairy roots.**

**Table S1. Summary of bisulfate sequencing and reads alignment**

| Tissues | Raw Reads | Clean Reads | BS Conversion Rate | Mapped reads | Genome coverage | coverage depth |
| --- | --- | --- | --- | --- | --- | --- |
| March_root | 86499159 | 84836367 | 99.62% | 41807361 | 88.18% | 16.25 |
| July_root | 67803025 | 66748243 | 99.63% | 31898985 | 87.47% | 14.94 |
| July_leaf | 67481438 | 65818905 | 99.56% | 39649308 | 89.47% | 18.81 |

**Table S9. Primers for quantitative real-time RT-PCR**

| **Gene name** | **Forward** | **Reverse** |
| --- | --- | --- |
| **AACT1** | AAAGCTCTGGAGCTCGGGCTTACA | TCCAAACCAGCATTCTTGAGTGCCT |
| **HMGS** | AGGACTGCAGCCTTCTTGCACCAG | TTCGCAATGGCCTTCTTGGCATAG |
| **HMGR1** | CAATAAGGAGGCTCCCGGATCCAA | ACTTCATGCTCGCAACATCCTTATT |
| **HMGR2** | CTCAACCTGCTGGGAGTCAAGGGA | TTGAGAGCTTAGTAACATCTTTGGAA |
| **MK** | TTCGTCTGCTGCTCTTTGTGTTGC | AGATGGCCTCCCATGGATCATCTT |
| **PMK** | TGCATGTCATCGCTCAAACTGCTC | TCAATTGGAATCCCTCGACCAGCA |
| **MDC** | AGTGCTTGCCGCAGCTTGTATGGT | CTGGTTTCAACCGTGTCACGCATT |
| **IDI1** | GCATCCAAATCCAGACGAAGTGCA | TTTCTCGACGTGGTCCCACCACTT |
| **FPPS** | GCGGGTGAGGACCTGGAGAAACAT | CAGGGCCTTTACAACCAGCCAAGAA |
| **GGPPS1** | CTGCATTGTTAGAGGCATCTGTAGTTT | AGCTTGGGATACGTGGTCTTGTCG |
| **DXS2** | CAGCGCCCATATTTCTCATTTCTTAT | GAAGACTGTCTTTTCCCCCACCAAT |
| **DXR** | TGGAGGCACCATGACCGGAGTTCT | GGGCCCACTGGTCGTAGTGGATGA |
| **MCT** | ACCTGAAATTTGCATTGCCTGGGAA | TCCTTGATTGTAGCCTTAGCAGGAA |
| **CMK** | CCAAGAGTGGTCGGGTGAGATTGG | GGGGCATGCCTCTTGTGGCTTAAT |
| **MDS** | TTGGCCACGGATTTGACCTTCATC | TGCCCTATATCTGGGAGCCCAAGC |
| **HDR2** | ATCGTATTGGGTTGACAGCGAGAAA | TATGCCGTTTGCAATACTTCATCGC |
| **CPS1** | CCACATCGCCTTCAGGGAAGAAAT | TTTATGCTCGATTTCGCTGCGATCT |
| **KSL1** | TGGAAACAGTGTGACCCTTCTGCT | GCTTGCATACAAATAACACCCAATCCT |
| **CYP76AH1** | CAATGCGCCAAAGAAGGATG | AGCATGAGATGGGTGAAGTG |
| **CYP76AH3** | AACCCCACGAGACATTC | CCGAGATGGACCGACA |
| **CYP76AK1** | CTACTCCACCCCGACAA | CGGATTCCTCCACGAT |
| **2OGD25** | CTGTGCAAGCAGTGAGGATG | GCATTTGGGATGGGCTGAAT |
| **CYP71D373** | AATCGCACCAACATCGTCTT | TCCATCGGCACAGGTTCAT |
| **CYP71D375** | AGTCCTTCCGCCACATAAGA | GTCATCGTTCCGCACTCTG |
| **TIIAS** | ACCGATCCTTGTTTCCTAAC | CCGATTTGCGTGGACTCTAT |
| **UBQ** | AGATGGGCGGACACTTGCTGATTA | ACTCTCCACCTCCAAAGTGATGGT |
